# Engineered an ultrasmall curcumin oral nanoformulation restores intestinal integrity and gut microbiota dysbiosis

**DOI:** 10.1016/j.mtbio.2025.102548

**Published:** 2025-11-12

**Authors:** Vivek Sharma, Prateeksha Prateeksha, Balwant Paliya, Sateesh Gupta, Sarvendra Singh, Anand Anunay, Sushil Agrahari, Shailendra Singh, Chandana Rao, Saroj Barik, Brahma Singh

**Affiliations:** a Herbal Nanobiotechnology Lab, Pharmacology Division, CSIR-National Botanical Research Institute, Lucknow, 226001, India; b Department of Biochemistry and Molecular Biology, Thomas Jefferson University, Philadelphia, PA, 19107, United States; c Key Laboratory of Bioactive Peptides of Yunnan Province, Kunming Institute of Zoology, Chinese Academy of Science, Kunming, Yunnan, 650000, China; d Department of Botany, Banaras Hindu University, Varanasi, 221005, India

**Keywords:** Carboxylated galactomannan, Ultrasmall nanoformulation, Intestinal epithelial barrier dysfunction, Gut inflammation, Gut microbiota, Secreted metabolites

## Abstract

Antibiotic therapy for bacterial infections often disrupts gut microbiota (GM) and intestinal integrity, inducing chronic inflammation and inflammatory bowel diseases. An effective therapeutic intervention is urgently needed to alleviate the aforementioned adverse effects of antibiotics. Curcumin (CUR) reveals great potential to restore intestinal integrity and GM dysbiosis due to its strong antiinflammatory and prebiotic effects. However, the weak solubility and stability of CUR result in limited bioavailability and a short half-life, which restricts its clinical uses. An ultra-small CUR oral nanoformulation was created using a carboxylated galactomannan (cGM), facilitated by hydrogen bonding between the phenolic hydroxyl groups of CUR and the carboxyl groups present on cGM. The developed nanoformulation increased CUR stability both *in vitro* and in vivo, extended its retention period in the gastrointestinal tract, and enhanced its permeability across the mucus layer and intestinal epithelium to improve oral bioavailability of CUR. The nanoformulation attained notable therapeutic efficacy in restoring intestinal epithelial barrier dysfunction and GM dysbiosis, as validated in an antibiotic-induced in vivo model. This research highlights the benefits of cGM in creating a very stable and ultrasmall nanoformulation for CUR, offering a promising oral nanoplatform for the delivery of CUR.

## Graphical abstract

**Figure:**
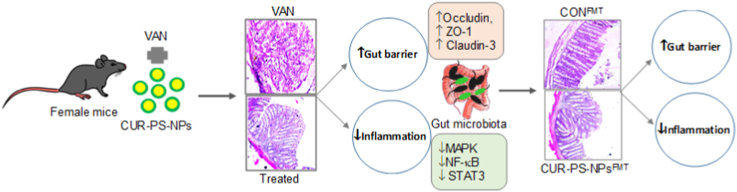

## 1 Introduction

Antibiotics are most commonly used to treat bacterial infections, and their global consumption rate has increased by 40 % over the past decade [1]. Antibiotic therapy often alters the functionality of occludin, ZO-1, and claudin-3, which are tight junction proteins, and can disrupt gut microbiota (GM) and intestinal integrity [2,3]. Emerging evidence has revealed that GM is essential to maintain the integrity of the gastrointestinal tract (GIT). GM dysbiosis leads to long-lasting inflammation in the gut, which can cause inflammatory bowel diseases like Crohn's disease and ulcerative colitis [[4], [5], [6], [7], [8]]. In previous investigations, including ours, the dietary polyphenol curcumin (CUR) has been shown to protect epithelial cells from inflammation and to increase the abundance of beneficial gut bacteria [[9], [10], [11], [12], [13]]. Given that CUR also improved GIT health [14], it is hypothesized that CUR could be a natural agent to prevent antibiotic-induced intestinal epithelial barrier dysfunction, inflammation, and GM dysbiosis [10,15,16].

The oral route offers convenience, ease of use, cost-effectiveness, and improved patient compliance, making it a preferred method for drug administration. Nonetheless, it is still difficult to provide CUR orally due to its low solubility, short half-life, and limited stability in the harsh conditions of the GIT [11,17,18]. Many studies have described various ways to deliver CUR in an oral form using nanoparticles, liposomes, nanoemulsions, and cyclodextrin complexes [19,20]. However, there is limited understanding about the bioavailability of CUR following oral treatment. Thus, it is vital to discover a more efficient oral delivery mechanism for CUR and to conduct additional research on its stability, retention duration in the GIT, absorption in the small intestine, and pharmacokinetic properties.

The use of nanocarriers to deliver therapeutic agents has become a pivotal approach in enhancing their effectiveness. Often, nanocarriers are prone to aggregate or destabilize in physiological environments, particularly in harsh GIT conditions [21,22]. Thus, overcoming challenges related to stability is crucial for their clinical translation. Plant-derived polysaccharide galactomannans demonstrate significant capacity for the encapsulation of drug agents, consequently enhancing both their stability and bioavailability. Galactomannans are nontoxic, biodegradable, and biocompatible biopolymers [23,24]. While there is consensus that the plant-derived galactomannan is the most suitable biomaterial for surface coating of nanoparticles [25], the lack of surface charge remains a challenge due to its moderate solubility, low stability at low pH in the GIT, moderate drug loading efficiency, fewer functionalization sites, and limited targeted delivery [26]. Accumulating evidence shows that negatively charged polysaccharides are generally more stable than positively charged ones due to their favorable electrostatic interactions with gastrointestinal ions, resistance to gut bacterial degradation, and stability across a broader pH range [[27], [28], [29]]. To create better nanoformulations of CUR, changing the chemicals in galactomannans might be a good way to improve their hydrophilicity, stability, bioactivity, and permeability in the GIT.

Numerous investigations have been conducted to improve the desired properties of polysaccharides by chemical modifications, among which carboxylation has been used as an effective method [30,31]. Carboxylated galactomannans (cGMs) not only exhibit greater hydrophilicity, drug loading efficiency, functionalization sites, and stability but also show superior adhesive and feasible targeted delivery than native galactomannans (nGMs) [31]. In addition, cGMs have exhibited diverse industrial applications due to their advantages of being nontoxic, bioadhesive, and biocompatible [32]. cGM creates flexible, colloidally stable formulations and preserves prebiotic fermentability of galactomannan, allowing GM-triggered colon administration, in contrast to alginate, which forms stiff ionically crosslinked gels with restricted fermentability. Chitosan derivatives, liposomes and micelles display various limitations, such as poor intestinal stability, rapid clearance, and limited mucus penetration. In contrast, our cGM@CUR-UsNF system integrates ultrasmall particle size, enhanced mucus adhesion, and sustained CUR release, thereby improving intestinal stability, absorption, and bioavailability. This addition distinctly emphasizes the innovation and benefits of the cGM@CUR-UsNF platform compared to traditional CUR nanocarriers.

The bioavailability of a drug molecule depends on how well it is absorbed, which is fundamentally related to its therapeutic properties. The translocation of CUR across the epithelial layer of the small intestine presents significant challenges. The mucus layer, intestinal epithelial cells (IECs), and the submucosal lamina propria (SMLP) are natural barriers in the small intestine that collectively exert substantial resistance against the infiltration of xenobiotics [[33], [34], [35]]. Ultrasmall nanomaterials (UsNMs) that are 1–5 nm in size is very promising for drug delivery systems because they can take up more drugs, pass through tissues easily, stay in the body longer, and release drugs in a targeted and controlled way [36,37]. UsNMs are smaller than the stated mesh-pore size, ranging from 10 to 200 nm in mucus [38] which can allow them to penetrate the mucus more easily and facilitate translocation across the intestinal epithelium and the SMLP [39]. These suggest that UsNMs could be applied to enhance the oral bioavailability of CUR for enhanced therapeutic effects.

**Scheme 1:**
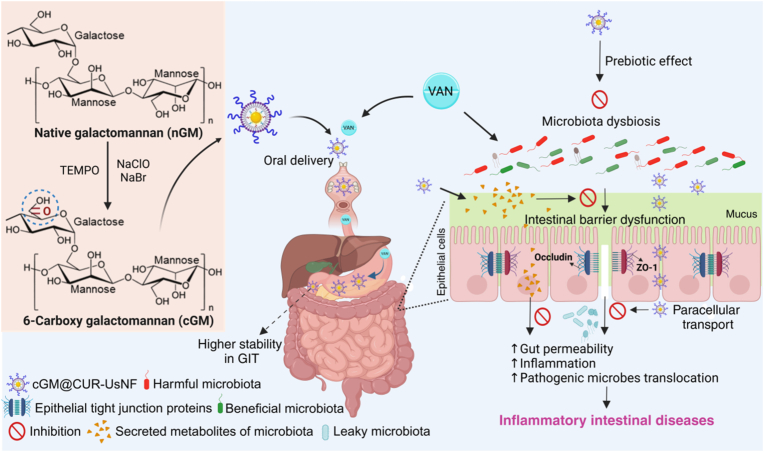
Schematic representation of the preparation of anionic-charged carboxylated galactomannan (cGM) through TEMPO-mediated oxidation at C-6 carbon of mannose/galactose units of galactomannan to develop a cGM-stabilized CUR oral nanoformulation (cGM@CUR-UsNF), which exhibits notable therapeutic ability to restore vancomycin (VAN)-induced intestinal epithelial barrier dysfunction, inflammation, and GM dysbiosis, validated in an in vivo model. Created with BioRender.com.

A significant advancement is necessary in the design and development of an oral nanoformulation of CUR using cGMs as a coating biomaterial that satisfies all criteria for the restoration of gut integrity. We suggest that this nanoformulation would have several benefits, such as better CUR stability *in vitro* and in vivo, extended retention time within the GIT, and improved permeability across the mucus barrier and IECs to improve how well CUR is absorbed when taken orally. We also expect that the CUR will work better to combat intestinal epithelial barrier dysfunction and gut inflammation, while also providing benefits like a prebiotic. Here, we developed an oral nanoformulation to restore gut integrity (Scheme 1). We meticulously designed the surface coating of these nanoparticles with an anionic-charged galactomannan that meets all the above-mentioned criteria. To change galactomannan, carboxyl (C=O) groups were added to it at C-6 using *N*-oxyl-2,2,6,6-tetramethylpiperidin (TEMPO) as a catalyst and this was studied using 1D and 2D NMR analyses. TEMPO selectively acts on the primary alcohol groups (–CH_2_OH) located at the C6 position of mannose and galactose units, transforming them into –COOH groups while leaving the secondary alcohols unaffected. These –COOH groups confer a negative charge on galactomannan, which interacts with the phenolic hydroxyl group of CUR via hydrogen bonding, leading to the preparation of a stable and bioavailable CUR oral nanoformulation, denoted as cGM@CUR-UsNF. The nanoformulation was characterized by transmission electron microscopy (TEM), dynamic light scattering (DLS), and Fourier transform infrared spectroscopy (FTIR) techniques. Researchers hypothesized that the nanoformulation would enhance GIT stability, retention time, and bioavailability. The effects of cGM@CUR-UsNF on restoring gut health were systematically tested using a vancomycin (VAN)-induced in vivo mouse model.

## 2 Materials and methods

### 2.1 Chemicals and reagents

Galactomannan (≥95 % purity; molecular weight, M_w_ = 2.0 × 10^5^ g mol^−1^; M/G ratio 4:1) of *Ceratonia siliqua* endosperm seeds was purchased from Sigma-Aldrich (48,230) (Bangalore, India). We procured VAN from HiMedia (Maharashtra, India) and the SOD estimation ELISA kit from R & G Systems Inc. (Minneapolis, USA). RIPA lysis buffer, protease inhibitor cocktail, Pierce ECL western blotting substrate, and primary antibodies against ZO-1 and occludin were procured from Thermo Fisher Scientific India Pvt Ltd. (Maharashtra, India). Kits for TUNEL assay, claudin-3, and caspase-3 were obtained from Elabscience (Houston, Texas, USA). Sodium hypochlorite, sodium bromide, 3-hydroxyisoquinoline (HIQ), 1,1′-dioctadecyl-3,3,3′,3′-tetramethylindotricarbocyanine (DiR), TEMPO, hematoxylin, eosin, methoxyamine hydrochloride, BSTFA, dimethylsulfoxide (DMSO), and FITC-dextran (4.4 kDa) were procured from Sigma-Aldrich Chemicals Pvt. Ltd. (Bangalore, India). Bradford Protein Assay kit was procured from Bio-Rad Laboratories (Hercules, CA). We obtained primary antibodies for I*κ*B*α*, STAT3, phospho-STAT3 (Tyr705), p65, phospho-p65 (Ser536), JNK, phospho-JNK (Thr183/Tyr221), caspase-3, cleaved-caspase-3 (Asp175), p38, phospho-p38 (Thr180/Tyr182), β-actin, and HRP-conjugated anti-goat secondary antibody from Abcam (Waltham, MA, USA). The institutional animal ethics committee was approved the study (1732/GO/Re/S/13/CPCSEA) and strictly followed the National Research Council's Guide for the Care and Use of Laboratory Animals.

### 2.2 Preparation of cGM

Galactomannan (2.5g) was solubilized in 250 mL of Milli-Q water (MQW) with continuous vigorous agitation (620 rpm). Subsequently, the addition of 2.5 mM TEMPO (0.2g) and 35 mM sodium bromide (2g) was conducted. The obtained galactomannan viscous solution was adjusted to pH 10 by adding 2 mM NaOH. A 4.5 % sodium hypochlorite solution (1.5g, 40 mM) was introduced to the galactomannan solution, followed by adjusting the pH to 10 through the addition of 2 mM NaOH, while maintaining continuous agitation (240 rpm) at 25 ± 1^o^C. The reaction was terminated after 24 h, by adding 5–7 drops of glycerol. The water-insoluble fraction was obtained through the filtration of the resulting product. Ethanol was added to the solution to precipitate it, and centrifugation was used for 10 min at 6000 rpm. To remove oligomers: low molecular weight galactomannan fragments or oligosaccharides formed due to partial depolymerization or hydrolysis during the oxidation process, the precipitated material was re-solubilized in water and subsequently subjected to dialysis using a Millipore ultrafiltration membrane of polyethersulfone (M_w_ cutoff: 10,000 g cm^−1^) within an Amicon cell outfitted with a reservoir containing MQW (with conductivity <3 μS m^−1^). When the filtrate conductivity fell below 10 μS m^−1^, the dialysis process was terminated and the cGM was obtained via freeze-drying (Labconco, USA). The cGM had a yield of approximately 65 %.

### 2.3 Synthesis of cGM@CUR-UsNF and nGM@CUR-UsNF

The cGM or nGM (300 mg) was dissolved in 5 mL of DMSO-MQW (1:1) at a temperature of 50^o^C in a reactor under a nitrogen protective environment and continuous agitation at 280 rpm. The pH was meticulously regulated at approximately 9.0 through the incremental addition of 2M NaOH. Subsequently, 100 mg (2.701 × 10^−4^ mol) of CUR (≥99.5 % purity; Sigma-Aldrich, India) was added dropwise to the reaction mixture. In this approach, DMSO-H_2_O, which acts as an essential universal solvent for the reaction milieu, was employed to directly promote the conjugation of cGM and CUR. After 3 h of vigorous stirring at 620 rpm, followed by sonication at 4^o^C for 2 min using a Fisher Scientific FS30D bath sonicator, the solution displayed an intense, clear, and transparent orange color, indicating the successful synthesis of cGM@CUR-UsNF and nGM@CUR-UsNF. The synthesized UsNFs were separated from the supernatant solution through centrifugation at 15,000 rpm for 20 min. Precipitates were redissolved in MQW, and the resulting solutions were subjected to the same treatment as the cGM preparation protocol. The obtained UsNFs were obtained via freeze-drying (Labconco, USA). The cGM@CUR-UsNF and nGM@CUR-UsNF had yields of approximately 90 % and 79 %, respectively.

### 2.4 Characterization of UsNFs

Bruker Biospin Avance 600 MHz and Bruker Biospin Avance 700 MHz spectrometers were used to record ^1^H NMR and ^13^C NMR spectra of the fabricated materials. Measurements of UV absorbance were carried out on an Evolution 201 spectrophotometer (Thermo Fisher Scientific, USA). FT-IR spectra of the materials were acquired using a Nicolet 6700 spectrophotometer (Thermo Fisher Scientific, USA). SEM images were obtained with a Quanta FEG-250 (Thermo Fisher Scientific, USA) at 30 kV. TEM images were taken with a JEM-1400 EX (JEOL, Japan) operated at 120 kV. DLS and zeta potential were measured with an Anton-Paar Litesizer 500 (Anton-Paar, India).

### 2.5 In vitro CUR release

The CUR release profiles of nGM@CUR-UsNF and cGM@CUR-UsNF were assessed using a membrane dialysis technique in SGF for the initial 2 h before transitioning to SIF. The chemical compositions of SGF and SIF were used as referenced [40]. Briefly, 1 mL of CUR formulations was positioned in a dialysis tubing (M_w_ of cut-off of 12 kDa) and placed in the release medium (20 mL). In a shaking incubator set to 100 rpm and 37 °C, samples of the medium were taken at specific intervals over a 24 h period, with an equal volume of fresh medium added each time. The amount of CUR that was released into the medium was quantified using reverse phase HPLC, as described in our previous study [13].

### 2.6 Cells and animals

Caco-2 human colon cancer cell line was provided by Procell Life Science & Technology Inc. (Wuhan, China), which was cultured in MEM medium (Procell, Wuhan, China). The THP-1 human acute monocytic leukemia cell line was provided by the Cell Bank of the Chinese Academy of Sciences (Shanghai, China), which was cultured in RPMI-1640 medium (GIBCO, Invitrogen Corporation, NY, USA). Meanwhile, 10 % (v/v) fetal bovine serum (Gibco, Vienna, Austria), 100 U/mL penicillin and 100 mg/mL streptomycin were added into the medium. The cells were maintained at 37 °C in a humidified atmosphere of 95 % air and 5 % CO_2_. Eight-week-old female C57BL/6 mice were obtained from CSIR-Central Drug Research Institute, India, and housed four in per cage under 50 % humidity and a 12-h light-dark cycle at 25 ± 1 °C for 7 days to acclimatize in the institutional animal house.

### 2.7 Stability of UsNFs

To examine the breakdown of nGM@CUR-UsNF and cGM@CUR-UsNF in SGF and SIF, Fӧrster resonance energy transfer (FRET) analysis was conducted. Formulations co-loaded with HIQ and NR (FRET pairs) were prepared by mixing galactomannan or cGM with HIQ (1 mg/mL) and NR (0.4 mg/mL), followed by sonication 150 W and 25 kHz in an ice-water bath and then filtered using a 0.1 μm membrane. Ultimately, the HIQ/NR@cGM was produced by exposing it to a 253.7 nm UV light at 36 W for 90 min. The control sample of HIQ/NR@GM was also prepared. The PS, PDI, and ZP of the prepared formulations were assessed through dynamic laser scattering. The LE was also determined. The prepared formulations were mixed with SGF or SIF in tubes. Following incubation at 37 °C on a shaking bed for various durations, samples were collected from the tubes and placed onto a 96-well plate. Fluorescence intensities were captured using a SYNERGY/HTX (BioTek, Germany) with the excitation at 510 nm and emission at 635 nm. The FRET ratio was determined using the formula FRET ratio = *I*_NR_/(*I*_NR_ + *I*_HIQ_), where *I*_HIQ_ and *I*_NR_ represented the fluorescence intensities of HIQ and NR at 510 nm and 635 nm, respectively, with an excitation wavelength of 450 nm. To assess the stability of UsNFs and bulk CUR in simulated intestinal fluid (SIF; pH 6.8, pancreatin 10 mg/mL, bile salts 5 mM) and simulated colonic fluid (SCF; fecal slurry, 10 % w/v, pH 7.0), samples were incubated at 37 °C with gentle agitation and collected at specified intervals. CUR retention was measured using HPLC following the separation of the released drug [41], while particle size was assessed through DLS analysis [13]. The stability of the polymer was evaluated using gel permeation chromatography and a reducing sugar assay. All experiments were conducted in triplicate.

### 2.8 In vitro permeation measurement assay

First, 5 × 10^4^ Caco-2 cells/well, as a gastrointestinal epithelium model were cultured in transwell plates (Thermo Fisher, India) with a pore size of 0.4 μm. Once the TEER values attained 500 Ω cm^2^, the cell monolayers were utilized for the subsequent experiments. Initially, mucin (10 mg/mL PBS) was introduced into the apical medium to mimic the occurrence of mucus, whereas PBS without mucin was utilized to represent the condition lacking mucus. Subsequently, IF (20 μL, 100 IU/mL) and 200 μL of CUR formulations (1 mg/mL) were introduced into the upper chambers. While incubating in a water bath at 37 °C, samples were taken from the lower chambers for analysis at various time intervals. A portion of the basolateral medium was taken out and examined using RP-HPLC to quantify the CUR content [13]. The *P*_app_ of CUR was determined using the method outlined in a previous study [34]. To analyze intracellular uptake, cells were gathered and underwent multiple freeze-thaw cycles prior to being lysed for 60 min. The concentration of CUR taken up by the cells was assessed using HPLC. Additionally, TEER measurements were recorded to evaluate the effect of CUR formulations on the monolayer's integrity. The baseline TEER readings for the Caco-2 cell layer were recorded. The apical media were then substituted with CUR formulations (200 μL). After 120 min, the CUR formulations in the upper chambers were removed and substituted with PBS. The TEER measurements of the Caco-2 cell monolayer were subsequently taken for various groups. To investigate the precise cellular uptake mechanisms of CUR formulations, Caco-2 cell monolayers were pre-incubated for 30 min with various inhibitory agents of endocytotic before being exposed to IF (20 μL, 100 IU/mL) and CUR formulations (200 μL) for 120 min. The *P*_app_ values for CUR were calculated.

### 2.9 Measurement of SOD and pro-inflammatory cytokines

The frozen samples of liver and ileum were homogenized in cold RIPA lysis buffer to obtain total proteins. Then we centrifuged the homogenates at 4 °C and 12,000×*g* for 12 min. We measured the protein content using a Bradford protein assay, following the kit instructions. Subsequently, SOD activity in the supernatants of hepatic and ileal tissues was measured following the kit instructions. For the measurement of pro-inflammatory cytokines, qPCR analysis was carried out to assess mRNA expressions of TNFα, IL-6, INFγ, and IL-1β. We used TRIzol reagent to isolate total RNA from the colon tissues. Reverse transcription was performed using an RNA-to-cDNA kit from Thermo Fisher (Waltham, MA, USA). The qPCR was conducted using Applied Biosystems SYBR Green Master Mix in a TagMan Applied Biosystems automatic RT-qPCR machine (Waltham, MA, USA). Primer sequences are provided in Table S1. We used GAPDH as an endogenous control. The relative expression levels of mRNAs were assessed using the 2^−ΔΔCt^ method.

### 2.10 Immunofluorescence analysis and TUNEL assay

We used paraformaldehyde (4 % w/v) to fix the colon sections and incubated them at 25 ± 1^o^C for 10 min. Then, the sections were permeabilized with 0.1 % (v/v) Triton X-100 and blocked in 10 % normal bovine serum albumin (Thermo Fisher, Maharashtra, India). The detection of claudin-3 and caspase-3 was performed using commercial kits according to the manufacturer's protocols. After mounting, sections were examined using a Leica DFC7000 T fluorescence microscope (Wetzlar, Germany) and analyzed with Image Pro Plus 6.0. The TUNEL assay was conducted in accordance with the instructions provided by the manufacturer. The random optical fields in each colonic tissue were selected and totaled for TUNEL^+^ cells per field using the above image analysis software.

### 2.11 Immunoblotting analysis

RIPA lysis buffer was used to prepare the colon tissue lysates, followed by centrifugation at 12,000×*g* for 20 min at 4 °C and kept for 10 min on ice. Protein content was measured, and 30 μg of protein was separated on a polyacrylamide gel at 100 V and transferred to polyvinylidene difluoride membranes at 230 mA for 120 min. The membranes were probed with primary antibodies for 12 h at 4^o^C, and then probed with a secondary antibody for 60 min at 25 ± 1^o^C. The membranes were developed using ECL substrate and visualized by a Thermo Fisher Chemiluminescence Imaging System (USA). We used ImageJ software to measure the intensities of protein bands. The expression of proteins was regulated to β-actin.

### 2.12 16S rRNA gene sequencing

Faecal microbial genomic DNA was isolated and quantified as described elsewhere [42]. Universal primers were employed to facilitate the amplification of the V3 and V4 domains of the 16S rRNA gene [43]. The obtained amplicons were normalized, aligned to create the sequencing library, and sequenced on an Illumina MiSeq (San Diego, USA). The raw data of 16S rRNA gene sequences were filtered based on high error probability (>0.01) and analyzed by QIIME version (1.9.1). We generated OTUs at an identity threshold of 97 % using UPARSE (version 7.0.1001). NMDS plots were developed using PAST version 2.17 [44]. Heatmap profiles were constructed using R Studio software version 3.6.1. To examine the differentially abundant biomarkers between the different groups, LEfSe analysis was performed. Spearman correlational studies were performed to assess the correlations between VAN-induced inflammation-related parameters and gut bacteria and metabolites.

### 2.13 Metabolome analysis

The extract of faecal samples (40 mg/ml methanol) was prepared as described in the previous method [45]. The obtained dry extract was derivatized with methoxyamine-HCl and BSTFA (Sigma-Aldrich, USA), followed by incubation for 1 h at 37^o^C. Subsequently, the supernatant (10 μl) obtained after centrifugation at 10,000×*g* for 12 min was injected into an Agilent GC-MS. The injector, transfer line, and ion source were set at 280^o^C, 150^o^C and 230^o^C, respectively. At the beginning, we set the oven temperature to 60^o^C, and then increased at 60 ^o^C/min for 2 min, followed by an increase at the rate of 10 ^o^C/min until reaching 300^o^C. We performed univariate statistical analysis using SPSS 16.0 by applying a paired *t*-test to compare metabolite contents between the VAN-induced and the treated groups.

### 2.14 Statistical analysis

Results were presented as means ± SEM. ANOVA, followed by Tukeyʼs test, was performed using GraphPad Prism (version 9.0) to examine the significant differences among all the treatments.

## 3 Results and discussion

### 3.1 Preparation and characterization of UsNFs

**Fig. 1:**
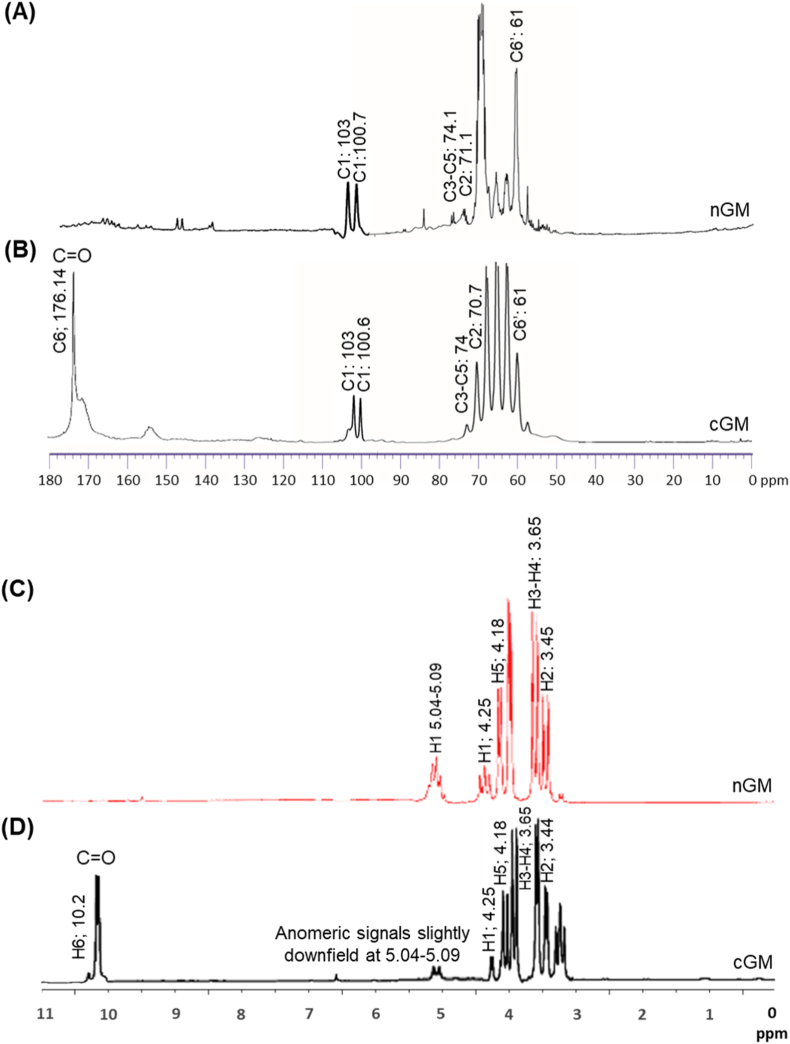
^13^C NMR spectra of the nGM (A) and cGM (B). ^1^H NMR spectra of the nGM (C) and cGM (D). C6′ indicates primary alcohols (-CH_2_OH) at C6 converted into carboxylic acids (-COOH).

The oxidation of nGMs facilitated by TEMPO was conducted as presented in Fig. S1 and subsequently characterized. The oxidation degree (OD) was 12 %, calculated using a formula [OD (%) = *I*_61_^(nGM)^ - *I*_61_^(cGM)^/*I*_61_^(nGM)^ X 100]. The cGM molar mass (M_w_) was determined to be 1.76 × 10^5^ g mol^−1^ using size exclusion chromatography. The lower M_w_ of cGM compared to nGM indicates that some glycosidic bonds were broken and the molecule was partially fragmented during oxidation [30]. TEMPO-mediated oxidation at 12 % introduces ∼0.53 mmol carboxyl groups g^−1^ of galactomannan. The ^13^C NMR spectra of the nGM showed a strong signal around 61 ppm, but this signal was weaker in cGM, which suggests that primary alcohols (-CH_2_OH) were turned into carboxylic acids (-COOH) (Fig. 1A and B). Concurrently, a new peak typically appears at 176.14 ppm, confirming the presence of carboxyl groups. The weaker signal at 61 ppm clearly shows that oxidation happened at the C-6 position, which is strong evidence that the galactomannan backbone was chemically changed [46,47]. The anomeric carbon (C-1) of nGM showed strong signals at 100.7 and 103 ppm, indicating that the glycosidic linkages were still present. After oxidation, the signals in this range decreased in cGM, which might mean that the structure changed because of broken chains or less organized crystals. ^1^H NMR spectra showed that the hydrogen signals (anomeric signals) at C-1 appeared between 5.04 and 5.09 ppm in nGM, but these signals were weaker in cGM, which suggests that nGM underwent some structural changes or partial chain degradation (Fig. 1C and D). Additionally, a new signal at 10.2 ppm from aldehydic proton confirm that the C-6 primary alcohol groups have changed, particularly due to the addition of carboxyl groups from the breakdown of primary alcohols. This corroborates that the hydroxyl groups at the C-6 carbon of galactomannans were successfully converted into carboxyl groups [48,49].

**Fig. 2:**
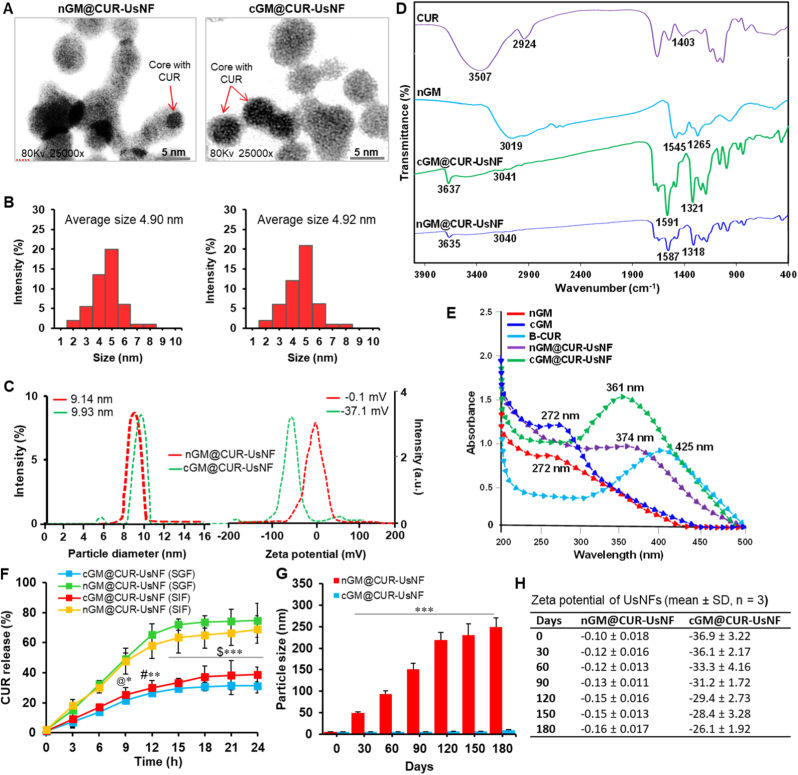
Characterization of UsNFs. (A) TEM images of nGM@CUR-UsNF and cGM@CUR-UsNF (scale bar 5 nm) and (B) their size distribution measured by TEM. (C) Size distribution and zeta potential of nGM@CUR-UsNF and cGM@CUR-UsNF measured by DLS. (D) FTIR spectra of CUR preparations. (E) UV–vis spectra of CUR preparations. (F) *In vitro* CUR release profile from nGM@CUR-UsNF and cGM@CUR-UsNF in both SGF and SIF conditions (*n* = 3). *p < 0.05, **p < 0.01, ***p < 0.001 compared with nGM@CUR-UsNF (SGF); **^@^***p* < 0.05, **^#^***p* < 0.01, **^$^***p* < 0.001 compared with nGM@CUR-UsNF (SIF). (G, H) Particle size and zeta potential of nGM@CUR-UsNF and cGM@CUR-UsNF at different time intervals (*n* = 3). ***p < 0.001 compared with nGM@CUR-UsNF.

We conducted 2D NMR analyses to confirm that TEMPO oxidation changed the C6 primary alcohols in the sugar units of cGM into carboxyl groups. The COSY spectrum of cGM shows clear connections between protons for mannose at *δ* 4.25 (H1) and 3.44 (H2), moving to *δ* 4.18 (H5), which connects with *δ* 10.18 (Table 1). For galactose, major peaks include *δ* 4.44 (H1) ↔ 3.65 (H3–H4), *δ* 4.19 (H5) ↔ 4.20 (H6), and *δ* 4.20 (H6) ↔ 10.20. The HSQC analysis of cGM shows clear signals for mannose at C1–H1 (100.6/5.08 ppm), C2–H2 (70.7/3.50 ppm), and C5–H5 (74.7/4.10 ppm), and for galactose at C1–H1 (101.9/5.04 ppm) and C5–H5 (72.4/4.20 ppm) (Fig. 2B). C6 signals are seen at 176.14 ppm for mannose and 176.15 ppm for galactose, with the absence of corresponding proton peaks indicating oxidation of primary alcohols to carboxylic acid groups. The HMBC spectrum of cGM reveals significant connections for mannose at H1/C1 (4.26/100.7 ppm), H2 (3.45), and H5/C6 (4.18/176.14 ppm) (Fig. 2C). Galactose exhibits strong signals at H1/C1 (4.25/100.8 ppm), H3–H4/C3–C5 (3.65/74.1 ppm), and H5, which connects to both C4 and C6 (4.18/176.15 ppm). The existence of *δ* ∼10 ppm (proton) and *δ* ∼176 ppm (carbon) signals without corresponding protons confirms selective TEMPO-mediated oxidation of C6 primary alcohols to carboxylic acids in both mannose and galactose. This targeted change was further supported by consistent COSY and HMBC correlations over sequential and long-range interactions. The 6-carboxy polysaccharides, not restricted to cGM notably exhibits higher water solubility and stability than their native forms [30,31].

**Table 1:** H-H COSY, HSQC and HMBC data of cGM and their correlations.

H-H-COSY			
	*Proton (δ, ppm)*	*Correlating Proton(s) (δ, ppm)*	*Assignment*
M	H1 (4.25)	H2 (3.44)	Sequential connectivity
	H5 (4.18)	*δ* 10.18	H6 → carboxylic acid (–COOH)
G	H1 (4.44)	H3–H4 (3.65)	Overlapping correlation
	H5 (4.19)	H6 (4.20)	
	H6 (4.20)	*δ* 10.20	H6 → carboxylic acid (–COOH)
**HSQC**			
	*Carbon (δ, ppm)*	*Proton (δ, ppm)*	*Assignment*

M	C1 (100.6)	H1 (5.08)	Anomeric
	C2 (70.7)	H2 (3.50)	
	C5 (74.7)	H5 (4.10)	
	C6 (176.15)	–	Carboxylic acid (–COOH)
G	C1 (101.9)	H1 (5.04)	Anomeric
	C5 (72.4)	H5 (4.20)	
	C6 (176.14)	–	Carboxylic acid (–COOH)
**HMBC**			
	*Proton (δ, ppm)*	*Carbon(s) (δ, ppm)*	*Assignment*

M	H1 (4.26)	C1 (100.7)	Anomeric
	H2 (3.45)	C3–C5 (74.0)	Overlapping
	H5 (4.18)	C6 (176.14)	C6 → carboxylic acid (–COOH)
G	H1 (4.25)	C1 (101.8)	
	H3–H4 (3.65)	C3–C5 (74.1)	Overlapping
	H5 (4.18)	C6 (176.15)	C6 → carboxylic acid (–COOH)

M, mannose; G, galactose.

The cGM@CUR-UsNF, containing CUR particles surface-coated with purified cGM, was successfully synthesized and confirmed. ^13^C NMR analysis showed that the hydroxyl groups of CUR were chemically attached to the carboxyl groups of cGM at 176.18 ppm (Fig. S2A), indicating that cGM@CUR-UsNF was created. ^1^H NMR further confirmed the formation of cGM@CUR-UsNF (Fig. S2B). The chemical shift observed for the anomeric signals matches the existence of terminal α-D-galactopyranose in the main chain, which is noticed at 4.83 ppm. Other small signals at 3.76 ppm and 3.77 ppm appeared to be α-D-galactopyranose and β-D-mannopyranose, respectively, at H-6 [50,51]. The cGM@CUR-UsNF also exhibited signals for aromatic hydrogens of CUR at 6.90–7.15 ppm [52] and at 9.70 ppm was likely due to a proton attached to the aromatic ring, specifically hydrogen on the phenyl group adjacent to the β-diketone moiety [53]. The results showed characteristic carbon and proton signals for both CUR and cGM, confirming the successful coating of cGM on CUR particles. In addition, the preparation of nGM@CUR-UsNF was also validated through ^13^C NMR (Fig. S3).

The morphological characteristics and dimensional distribution of UsNFs were examined using TEM and DLS techniques. The nGM@CUR-UsNFs exhibited a slightly translucent and irregular shape, while the cGM-UsNF loaded with CUR displayed a spherical form with a consistent size distribution (Fig. 2A). Additionally, the CUR-filled core−shell structure of cGM in the cGM@CUR-UsNFs was detected, confirming the effective coating of cGM onto CUR particles. The size distribution graphs showed that the particle sizes of nGM@CUR-UsNF and cGM@CUR-UsNF were about 4.90 ± 0.16 and 4.92 ± 0.19 nm, respectively (Fig. 2B), which were much smaller than the hydrodynamic particle size measured by DLS (Fig. 2C). Moreover, the zeta potential of nGM@CUR-UsNF was approximately −0.1 ± 0.01 mV and became −37.1 ± 2.9 mV after being CUR coated with cGM for cGM@CUR-UsNF (Fig. 2C). The changes might be related to the CUR particles being covered by cGM, which gives them a stronger negative charge [25].

The infrared spectra of the UsNFs were recorded using FTIR to study the surface chemistry of the UsNFs. In Fig. 2D, the cGM@CUR-UsNF showed a characteristic peak related to the stretching vibrations of hydroxyl groups (-OH) present in the sugar units of nGM at 3637 cm^−1^, which is indicative of GM in the UsNF framework. In both cGM@CUR-UsNF and nGM@CUR-UsNF, peaks emerged at around 3041, 1591, and 1321 cm^−1^, exhibiting the aromatic C-H stretching vibrations from the benzene rings of CUR [54], the stretching vibrations of the conjugated double bonds (C=C) between the aromatic rings and the diketone structure in CUR [55], and the C-O stretching vibrations of the phenolic hydroxyl group of CUR [56], respectively. These results indicate that the interaction between CUR and cGM was facilitated by hydrogen bonding occurring between the phenolic hydroxyl groups of CUR and carboxyl groups present on cGM (Fig. S4A and B).

The UV–vis spectra showed that unique absorption peaks were found at 374 and 425 nm for nGM@CUR-UsNF and CUR, respectively [57]. Notably, a more intense peak appeared at 272 nm after GM modification into cGM (Fig. 2E), which was similar to the distinctive absorption peak of nGM [58]. A new peak appeared at 361 nm after CUR particles were coated with cGM, indicating the absorption peaks are cross-fused for cGM@CUR-UsNF.

The loading efficiency (LE) and loading capability (LC) of both nGM@CUR-UsNF and cGM@CUR-UsNF were evaluated (Table S2). Both nGM@CUR-UsNF and cGM@CUR-UsNF exhibited comparable LE (∼70 %) and LC (∼11 %), indicating similar CUR encapsulation performance between the cGM and nGM. The release kinetics of CUR from the UsNFs was also measured for 24 h in simulated gastric fluid (SGF; pH 2.5) and simulated intestinal fluid (SIF; pH 7.0) at 37 ± 1 °C (Fig. 2F). The cGM@CUR-UsNF demonstrated a significantly inhibited release profile, with a diminished cumulative release of CUR at 24 h in SGF and SIF, which was reduced by 2.4-fold and 1.8-fold (p < 0.001) compared to nGM@CUR-UsNF, respectively. The cGM@CUR-UsNF displayed markedly enhanced stability, maintaining particle size and zeta potential after being kept for 24 h in both SGF (pH 2.5) and SIF (pH 7.0) conditions, whereas nGM@CUR-UsNF showed pronounced aggregation and reduced surface charge (Table S3), signifying the superior colloidal stability of the cGM@CUR-UsNF. The results demonstrate that cGM endowed cGM@CUR-UsNF with the capacity to function as a pH buffer. Additionally, the stability of UsNFs, which were dissolved at a concentration of 2 mg/mL in MQW, was also tested over 180 days at a temperature of 37 ± 1 °C (Fig. 2G and H). The cGM@CUR-UsNF demonstrated no significant alteration in its visual characteristics; conversely, nGM@CUR-UsNF exhibited some precipitation at 180 days. In parallel, a progressive increase in the particle size of nGM@CUR-UsNF was recorded over the 180-day period, whereas cGM@CUR-UsNF displayed no significant changes in both particle size (Fig. 2G) and zeta potential (Fig. 2H). The autoxidation, hydrolysis, and degradation-induced content loss of CUR is a significant issue. The free-CUR exhibited over 73 % degradation during 180 days of storage at a temperature of 37 ± 1 °C (Fig. S5). The nGM@CUR-UsNF mitigated CUR loss to a degree, retaining 51 % of the overall CUR content. The cGM@CUR-UsNF constituted about 83 % of the total CUR, markedly surpassing the free-CUR (27 % remaining) and nGM@CUR-UsNF (51 % remaining). Collectively, cGM demonstrates significant potential for effective oral administration of CUR.

### 3.2 *In vitro* translocation of CUR

**Fig. 3:**
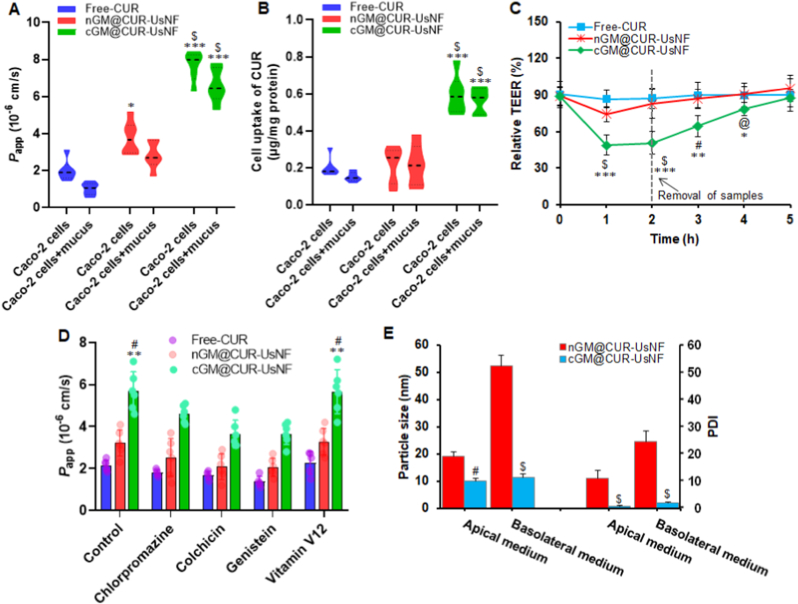
Translocation of CUR across the mucus layer and caco-2 cell monolayer (*n* = 6). (A) *P*_app_ values of free-CUR and UsNFs in Caco-2 cell monolayer with or without mucus at 2 h. (B) Cellular uptake of free-CUR and UsNFs in Caco-2 cell monolayer with or without mucus at 1 h. (C) Relative TEER changes of the Caco-2 cell monolayer after treatment by free-CUR and UsNFs (*n* = 3). (D) *P*_app_ values of free-CUR and UsNFs in Caco-2 cell monolayer under different inhibitory conditions. (E) Particle size distribution of UsNFs in different media (*n* = 3). *p < 0.05, **p < 0.01, ***p < 0.001 compared with free-CUR; **^@^***p* < 0.05, **^#^***p* < 0.01, **^$^***p* < 0.001 compared with nGM@CUR-UsNF.

An *in vitro* transwell system with either mucus or a Caco-2 cell monolayer was used in the upper chamber to assess how well cGM@CUR-UsNF and nGM@CUR-UsNF can pass through small epithelial cells. We studied the permeability coefficient (*P*_app_) of CUR using the Caco-2 cell monolayer to assess its translocation across IECs (Fig. 3A). The cGM coating on cGM@CUR-UsNF increased the movement of CUR by 3.8 times compared to free-CUR and 2 times compared to nGM@CUR-UsNF. The cGM@CUR-UsNF demonstrated a remarkable ability to move CUR through the mucus, likely because it does not interact with the mucus due to its negative charge, small size, and stability. The mechanisms of transepithelial transcytosis and paracellular transport are likely to play a role in the permeation of CUR. To elucidate the significance of the cellular pathway in the permeation of CUR, we conducted an assessment of Caco-2 cellular uptake (Fig. 3B). The cGM@CUR-UsNF markedly enhanced the cellular uptake of CUR when compared with free-CUR. Even though the mucus layer made it hard for CUR to enter the cells, cGM@CUR-UsNF allowed much more CUR to be taken up by the cells than free-CUR and nGM@CUR-UsNF. It was supposed that cGM-mediated cellular uptake could facilitate CUR penetration through a paracellular route.

We measured how substances move between cells by checking the transepithelial electrical resistance (TEER) (Fig. 3C). The cGM@CUR-UsNF applied to the apical side of cell monolayers quickly decreased the TEER and then slowly returned to baseline after the samples were removed, while free-CUR and nGM@CUR-UsNF kept the TEER steady, demonstrating the immediate and reversible disruption of tight junctions. Furthermore, the mechanisms through which cGM@CUR-UsNF cross the Caco-2 cell monolayer were systematically examined (Fig. 3D). The *P*_app_ values for cGM@CUR-UsNF went reduce after treatment with chlorpromazine, colchicine, and genistein, indicating that clathrin, caveolin, and micropinocytosis-mediated endocytosis helped in the uptake of UsNFs. Vitamin B12 is known to bind with intrinsic factor, helping it move through the intestinal lining by using specific receptors in the ileum [59]. Adding vitamin B12 did not change the *P*_app_ value of cGM@CUR-UsNF, which shows that paracellular transport helped cGM@CUR-UsNF get through, likely because the particles are very small and stable.

To assess the integrity of UsNFs after they entered the cells, we measured the particle size (PS) and polydispersity index (PDI) of nGM@CUR-UsNF and cGM@CUR-UsNF on both sides of the Caco-2 cell layer after 2 h. No significant changes were observed in the PS and PDI of cGM@CUR-UsNF, but the nGM@CUR-UsNF showed higher PS and PDI (Fig. 3E), indicating that it became less stable. The presence of mucins [60], enzymes, and endogenous phospholipids [61] might compromise the stability of nGM@CUR-UsNF by getting in between the CUR and polysaccharide coating during absorption in the GIT, indicating that these substances attaching to the nanoparticle surface could change the shape of nGM@CUR-UsN. The retention of cGM@CUR-UsNF integrity could be closely associated with the presence of negative charge on the cGM coating, which somewhat repels other negatively charged molecules, allowing it to maintain its position and resist being degraded or removed by mucins. In addition, the carboxylation could make the cGM coating less susceptible to enzymatic cleavage by altering its conformation or creating a steric hindrance that stops enzymes from reaching important areas that need to be cut.

### 3.3 Retention of UsNFs in the GIT and pharmacokinetics (PK) of CUR

Prior to assessing the retention period of UsNFs in the GIT, the stability of nanoformulations in different simulated environments was examined using HIQ and nile red (NR) as the Fӧrster resonance energy transfer (FRET) reagent pair [34]. Through FRET, energy from the donor-HIQ can be transferred to the receptor-NR, leading to the emission from NR while the emission from HIQ decreases. The nGM-UsNF and cGM-UsNF were embedded with HIQ and NR (Table S4) and measured the FRET ratio after 12 h incubation in SGF and SIF media (Fig. S6A and B). The HIQ/NR@cGM-UsNF showed much better stability than HIQ/NR@nGM-UsNF in SGF and SIF media, which is indicated by the NR fluorescence and FRET ratio. Additionally, the stability of UsNFs were also assessed in simulated intestinal fluid (SIF, pancreatin + bile) and simulated colonic fluid (SCF, fecal slurry). In SIF, cGM@CUR-UsNF demonstrated superior stability, maintaining approximately 70 % of CUR after 24 h, in contrast to around 40 % for nGM@CUR-UsNF and less than 10 % for B-CUR (Fig. S7A). Particle size analysis indicated that cGM@CUR-UsNF maintained relative stability (9.5–13 nm over 24 h), while nGM@CUR-UsNF experienced rapid aggregation, surpassing 125 nm within the same time frame (Fig. S7C). In SCF, both formulations exhibited progressive degradation; however, cGM@CUR-UsNF demonstrated a slower rate of CUR loss, retaining approximately 45 % at 24 h compared to 18 % for nGM@CUR-UsNF (Fig. S7B). Additionally, cGM@CUR-UsNF showed more regulated particle growth, remaining at or below 20 nm after 24 h (Fig. S7D). Polymer molecular weight analysis consistently indicated a gradual depolymerization of cGM, reaching 60 % of the initial molecular weight at 24 h, while nGM exhibited a more rapid degradation, reducing to 18 % of the initial molecular weight at the same time point (Fig. S7E). The results demonstrate that carboxylation markedly improves particle stability under intestinal conditions and facilitates controlled, enzyme-responsive degradation in colonic fluid, thereby affirming its appropriateness for intestinal transit and colonic release.

We first determined the doses of UsNFs and free-CUR in eight-week-old female C57BL/6 mice before systematically evaluating the retention of UsNFs in the GIT and PK of CUR. The data on body and liver weight indicated that UsNFs and free-CUR were well tolerated at doses of up to 50 mg/kg, whereas a dose of 75 mg/kg resulted in adverse effects (Table S5). The groups administered 25 mg/kg and 50 mg/kg of nGM@CUR-UsNF, cGM@CUR-UsNF, and free-CUR exhibited no significant differences in final body weight or relative liver weight when compared to the normal control, suggesting favorable systemic safety. The 75 mg/kg dose groups of UsNFs and free-CUR demonstrated significant reductions in both body weight and liver weight, indicating potential toxicity or metabolic stress at elevated concentrations. Consequently, 50 mg/kg was identified as the optimal dosage for subsequent in vivo therapeutic assessment of both UsNFs and free-CUR, achieving a balance between efficacy potential, safety, and physiological tolerance.

The residence time of DiR-decorated UsNFs in the GIT was investigated by applying an optical imaging approach. Following oral treatment, the NIR signal from DiR was detected at specific periods, and the radiant signals in the different organs were measured (Fig. S8A-E). Free DiR demonstrated quick removal via feces from the GIT across all groups. The cGM@DiR-UsNF stayed in the duodenum and jejunum longer than nGM@DiR-UsNF within 2 h, suggesting that cGM helps the small intestinal cells take in the substance, which may slow down its movement through the digestive system. The results indicated that cGM@DiR-UsNF significantly improved the paracellular translocation of DiR through epithelial cells, a crucial factor for attaining enhanced bioavailability.

**Fig. 4:**
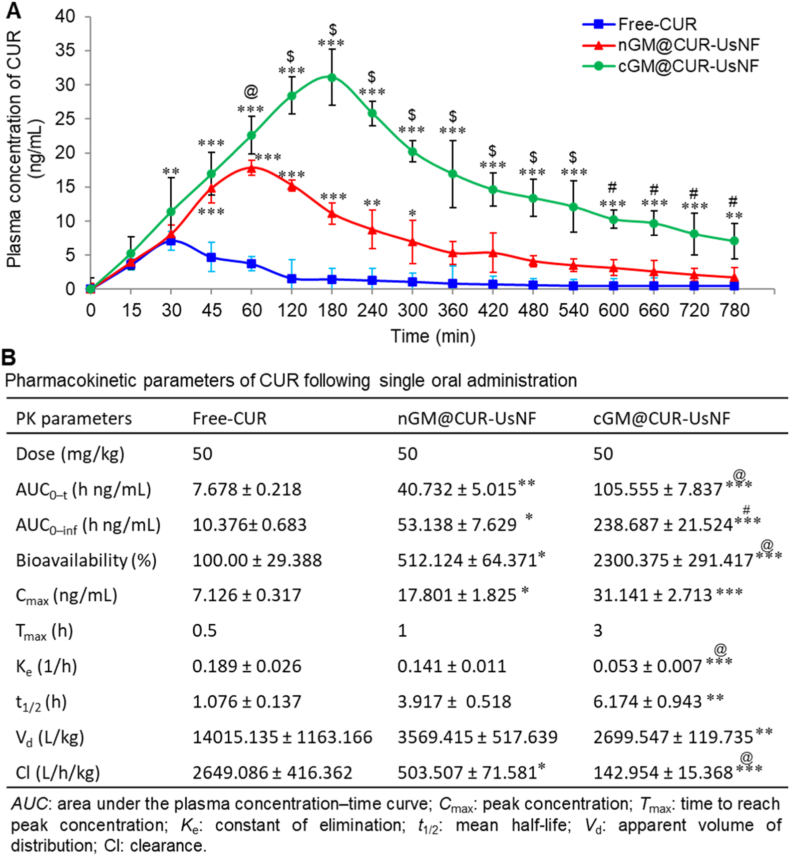
Pharmacokinetics of CUR. (A) Plasma drug concentration−time curves and (B) major pharmacokinetic parameters of CUR following oral administration of free-CUR and UsNFs at a dose of 50 mg/kg in C57BL/6J mice (*n* = 6). *p < 0.05, **p < 0.01, ***p < 0.001 compared with free-CUR group; **^@^***p* < 0.05, **^#^***p* < 0.01, **^$^***p* < 0.001 compared with nGM@CUR-UsNF group.

The most crucial assessment criterion for UsNFs is the bioavailability of CUR aqueous following oral administration. We looked at how the levels of free-CUR, nGM@CUR-UsNF, and cGM@CUR-UsNF changed in the blood over time after taking them orally (Fig. 4A), and we figured out the main PK factors of CUR (Fig. 4B). The maximum plasma concentration (*C*_max_) in the free-CUR group reached around 7.13 ng/mL, followed by a swift elimination phase. nGM@CUR-UsNF and cGM@CUR-UsNF enhanced the area under the concentration-time curve (AUC0-∞) of CUR by 5.3 and 13.7 folds, respectively, compared to free-CUR. Nevertheless, nGM@CUR-UsNF failed to increase the *C*_max_ of CUR. The cGM@CUR-UsNF showed the uppermost *C*_max_ and most prolonged half-life (*t*_1/2_) of CUR compared to the free-CUR and nGM@CUR-UsNF groups. As a result, the bioavailability of CUR in cGM@CUR-UsNF was over 20 times greater than that of the free-CUR group. The bioavailability of cGM@CUR-UsNF was more than 2-fold and 5-fold higher than an organogel-based nanoemulsion [62] and PLGA nanoparticles [63] of CUR, respectively. The enhanced stability, prolonged retention, and ultrasmall size of the cGM@CUR-UsNF may support the paracellular movement of CUR, which is essential for preventing dysfunction in the intestinal epithelial barrier and reducing gut inflammation.

### 3.4 Therapeutic effects of cGM@CUR-UsNF on VAN-induced intestinal epithelial barrier dysfunction and gut inflammation

**Fig. 5:**
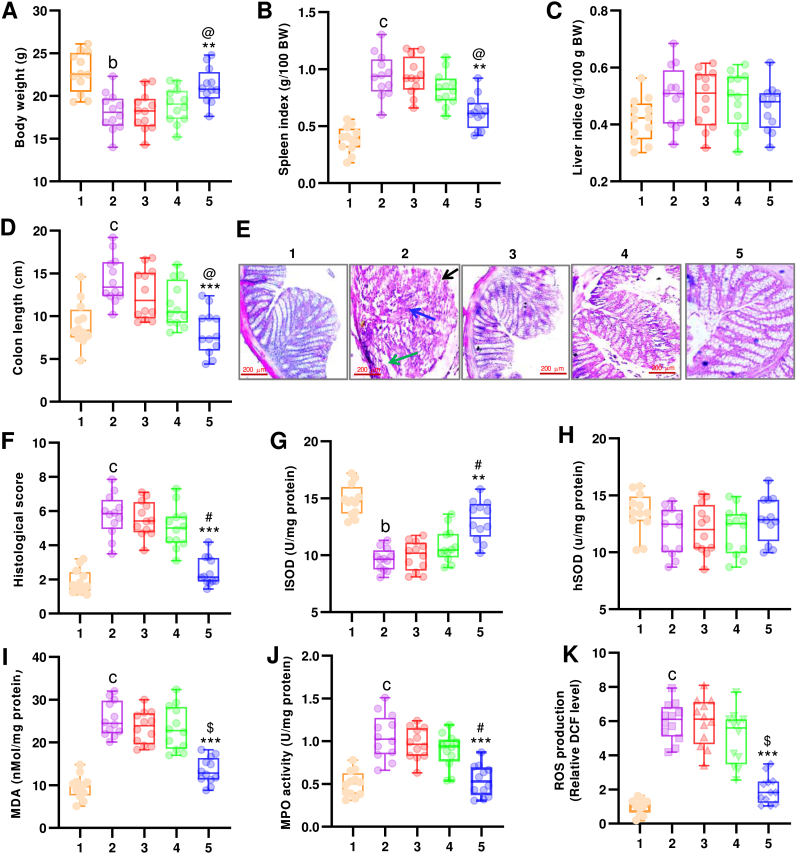
Impact of UsNFs on VAN-induced inflammatory parameters and oxidative stress. VAN-induced (20 mg/ml in the drinking water for 10 days) mice were treated with test samples (50 mg/ml) for another 10 days. On day 21, mice were sacrificed for the examination of indicated parameters (*n* = 12). (A) Body weight, (B) spleen index, (C) liver index, (D) colon length (CL), (E) H&E-stained colon sections, (F) histological score; green arrow shows damaged crypt, black arrow shows destructed luminal surface, blue arrow shows lymphocytes infiltration, (G) iSOD activity, (H) hSOD activity, (I) MDA content, (J) MPO activity, (K) ROS production. 1, CON; 2, VAN; 3, free-CUR; 4, nGM@CUR-UsNF; 5, cGM@CUR-UsNF. ^b^*p* < 0.01, ^c^*p* < 0.001 compared to CON group; **p < 0.01, ***p < 0.001 compared with VAN group; **^@^***p* < 0.05, **^#^***p* < 0.01, **^$^***p* < 0.001 compared with nGM@CUR-UsNF group.

To evaluate the therapeutic potentials of cGM@CUR-UsNF, a VAN-induced C57BL/6J mouse model was created, and these mice were given 20 mg/ml VAN in their drinking water for a duration of 10 days. This was succeeded by a daily oral administration of 50 mg/kg UsNFs of CUR for another 10 days, whereas the control group (CON) received standard rodent feed and water (Fig. 5A). VAN, a glycopeptide antibiotic used to treat infections caused by Gram-positive bacteria, can indeed induce intestinal inflammation and GM dysbiosis [64]. The weight fluctuations of the mice were documented throughout the treatment (Fig. 5A). The administration of VAN resulted in a pronounced reduction in the body weight of mice, with this reduction being statistically relevant when compared to the CON group (*p* < 0.01). The cGM@CUR-UsNF treatment resulted in no loss of body weight, which was significantly (*p* < 0.01) different from that of VAN, nGM@CUR-UsNF, and free-CUR groups. The spleen is a key organ for the regulation of inflammation-related immune responses, and an increase in splenic index is associated with inflammatory responses [65]. The splenic index in the VAN-induced group was 2.3-fold greater than the CON group (Fig. 5B, *p* < 0.001). The cGM@CUR-UsNF superiorly antagonized these elevations as compared to free-CUR and nGM@CUR-UsNF (*p* < 0.05). No significant alterations in liver indices among the treatments were observed (Fig. 5C).

The size of the colon greatly affects how well the GIT works, including its ability to absorb nutrients, maintain a healthy microbiome, and support the immune system [66]. We recorded colon length, since VAN administration for 10 days had resulted in a longer length in the experiment conducted by the earlier workers. We found that the average length of the colon in the mice treated with VAN was 21.9 % longer than in the control group (*p* < 0.01), but the treatment with cGM@CUR-UsNF significantly reduced the colon length compared to both the VAN group (*p* < 0.01) and the nGM@CUR-UsNF group (Fig. 5D, *p* < 0.05). However, we observed no significant decrease in the free-CUR and nGM@CUR-UsNF-treated groups. To assess the protective effect of cGM@CUR-UsNF on the colon, we performed histological analysis (Fig. 5E), which revealed that the colon of the healthy control group exhibited undamaged epithelial structure with no lymphocyte infiltration. On the other hand, the colon in the VAN group showed abnormal crypt shapes, a damaged surface, fewer goblet cells, and more lymphocyte infiltration. Treatment with cGM@CUR-UsNF clearly restored the histological structure of the colon, as evidenced by undamaged crypts, an intact epithelial layer, higher goblet cell counts, and almost no lymphocyte infiltration. We also obtained a lower histological score for the cGM@CUR-UsNF group compared to the VAN (*p* < 0.001) and nGM@CUR-UsNF groups (Fig. 5F, *p* < 0.01). Emerging evidence has demonstrated that the antibiotic can induce the formation of reactive oxygen species (ROS) in animal and human tissues [67]. Oxidative stress and inflammation play a major role in the development of inflammatory intestinal disorders by reducing the endogenous antioxidant system [68]. We measured the activity of ileal superoxide dismutase (iSOD) and hepatic SOD (hSOD) (Fig. 5G). As expected, the VAN group exhibited significantly reduced iSOD activity (*p* < 0.01); but no significant alteration was detected in hSOD activity (Fig. 5H), suggesting the generation of oxidative stress by VAN in the intestine. The cGM@CUR-UsNF effectively restored the activity of iSOD (*p* < 0.01). It was almost comparable to the activity of the CON group. In contrast, treatment with cGM@CUR-UsNF and free-CUR did not restore the activity of iSOD. The anti-inflammatory and antioxidant activities of nano-CUR had been confirmed in our earlier study [69] and also by other researchers [[70], [71], [72]]. Excessive reactive oxygen species (ROS) in VAN-induced colitis damaged lipids, resulting in elevated malondialdehyde (MDA) levels, a critical indicator of oxidative stress in intestinal damage [73,74]. In the VAN-induced group, MDA levels escalated by around 2.5 times, but cGM@CUR-UsNF therapy reinstated them to nearly normal levels (Fig. 5I). Conversely, free-CUR and nGM@CUR-UsNF only partially diminished MDA buildup. Likewise, cGM@CUR-UsNF significantly reduced myeloperoxidase (MPO) activity to baseline levels, surpassing the other treatments (Fig. 5J). MPO activity in colon tissues indicates oxidative stress and the infiltration of inflammatory cells [75]. Fluorescence imaging using DCFH-DA consistently demonstrated that cGM@CUR-UsNF more effectively scavenged intracellular ROS than free-CUR and nGM@CUR-UsNF in VAN-induced colon tissues (Fig. 5K). The aforementioned findings demonstrated that cGM@CUR-UsNF's exceptional ROS scavenging capability could successfully shield colon cells from oxidative stress damage and preserve their regular cellular activity.

**Fig. 6:**
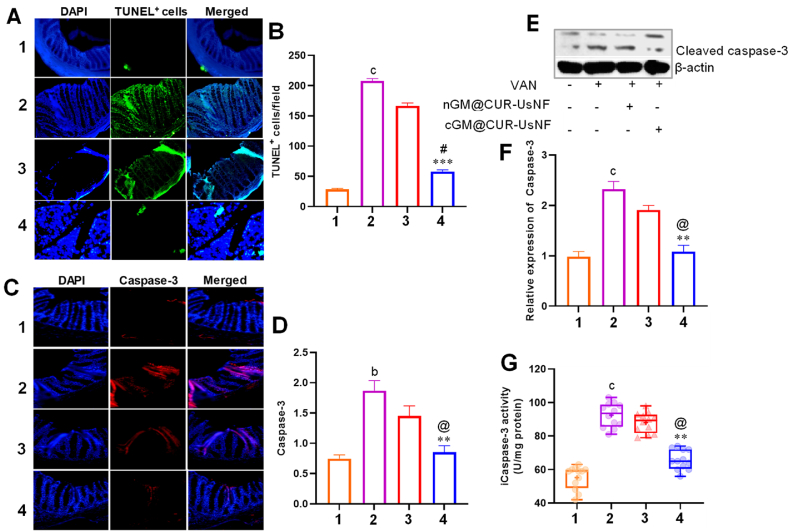
Impact of UsNFs on VAN-induced apoptosis in the colon tissues (*n* = 12). (A) TUNEL experimentation; (B) quantification of TUNEL^+^ cells; (C) immunohistochemistry of caspase-3; (D) quantification of Caspase-3; (E) immunoblotting analysis of cleaved-caspase-3; (F) relative expression of cleaved-caspase-3; (G) iCaspase-3 activity. Scale bars 50 μM. ^b^*p* < 0.01, ^c^*p* < 0.001 compared to CON group; **p < 0.01, ***p < 0.001 compared with VAN group; **^@^***p* < 0.05, **^#^***p* < 0.01 compared with nGM@CUR-UsNF group. 1, CON; 2, VAN; 3, nGM@CUR-UsNF; 4, cGM@CUR-UsNF.

A key role of the gut epithelial tight junction barrier is to protect the host against luminal antigens and exogenous pathogens by forming a biological barrier and triggering the immune system, resulting in gut homeostasis [76,77]. However, antibiotics disrupt this barrier [78], which increases the risk of several diseases, including inflammatory bowel disease, intestinal ischemia, shock, and severe burns [79]. As illustrated in Fig. S9A, VAN greatly (*p* < 0.001) made the intestines more permeable to 4.4 kDa FITC-dextran compared to the CON group. Treatment with cGM@CUR-UsNF significantly reduced this increase, as compared to VAN (*p* < 0.01) and nGM@CUR-UsNF groups (*p* < 0.05). The levels of occludin, ZO-1, and claudin-3, which are key members of the tight junctions in the gut lining [77,79], were measured using immunoblot (Fig. S9B) and RT-PCR analyses (Fig. S9C and D). When compared with the CON group, results showed a decrease in expressions of ZO-1 and Occludin in the VAN group, resulting in disrupted gut paracellular permeability and induced loss of intestinal barrier function. The VAN group exhibited a downregulated expression of ZO-1 and Occludin, whereas cGM@CUR-UsNF treatment significantly upregulated the expression of ZO-1 and Occludin. Next, immunofluorescence analysis of mice colon tissues was performed to evaluate the localization of claudin-3 (Fig. S9E and F). We observed reduced colon distribution of claudin-3 in the VAN group, while cGM@CUR-UsNF treatment significantly reversed this reduction and touched the level examined in the CON group (*p* < 0.001). Since expressions of intestinal tight junction proteins (iTJPs) are key defining factors for gut barrier function, higher expression of iTJPs offers a further reason for the superior protective effects of cGM@CUR-UsNF compared to nGM@CUR-UsNF. Given that the aberrant apoptosis induced by antibiotics has been reported to alter the permeability of the gut barrier, a key event for inducing gut barrier dysfunction [80], the level of apoptosis in colon tissues was measured after treatment using a TUNEL assay (Fig. 6A and B). The CON group rarely showed TUNEL^+^ cells in their colonic epithelia. In contrast, VAN mice had significantly elevated TUNEL^+^ cells (*p* < 0.001). The cGM@CUR-UsNF treatment significantly reduced TUNEL^+^ cells compared to the VAN (*p* < 0.001) and nGM@CUR-UsNF groups (*p* < 0.01).

It has been reported that dietary phytochemicals might help improve gut health by controlling the activity of caspase-3, [81], which is important for cell death [82]. CUR might exert its positive effects on gut integrity via regulation of caspase-3 activity. The expression level of caspase-3 which is intimately associated with gut barrier integrity, was studied using immunofluorescence (Fig. 6C and D) and immunoblot analysis (Fig. 6E and F). The expression of caspase-3 in the VAN group was significantly higher than those in the CON group. The administration of cGM@CUR-UsNF led to a much larger decrease in caspase-3 levels compared to nGM@CUR-UsNF (*p* < 0.01), improving gut caspase-3 activity (Fig. 6G) and lowering the number of TUNEL^+^ cells. Several comparable investigations have demonstrated that CUR attenuates apoptosis in intestinal epithelial cells [83], post-bile duct ligation rat mucosa [84], and colon mucosa of ulcerative colitis mice [85]. A recent study found that patients with repeated antibiotic treatments had a 55 % higher risk of inflammatory bowel disease (IBD) due to increased production of pro-inflammatory cytokines compared to those who did not use antibiotics [86]. Numerous studies have shown that antibiotic treatment changes the levels of inflammatory cytokines in the colon [66], ileum, and liver [87]. The anti-inflammatory effects of CUR have been well-studied, so the levels of inflammatory proteins in the colon tissues of mice were measured after treatment using immunoblot analysis (Fig. S10A–J). VAN markedly induced inflammation, as evidenced by strong I*κ*B*α* degradation, triggered the NF-*κ*B (p65) phosphorylation (*p* < 0.01 to 0.001), and increased the expression of p-STAT3/STAT3, indicating the activation of the NF-*κ*B/STAT3 pathways. It is well-established that NF-*κ*B/STAT3 pathways are associated with antibiotic-induced gut inflammation [66,86]. Various stimuli activate STAT proteins, triggering the gene expression of numerous cytokines in the nucleus [88]. Blocking the activation of NF-κB/STAT3 showed promising results for treating inflammatory gut diseases [89,90]. The cGM@CUR-UsNF significantly inhibited the phosphorylation of I*κ*B*α* (*p* < 0.01), NF-κB (*p* < 0.05), and STAT3 (*p* < 0.01) as compared to the VAN group, terminating the inflammatory reactions induced by VAN in mice. JNK and p38/MAPK pathways represent the subgroups of the MAPK signaling that control the expression of NF-*κ*B that results in increased inflammatory responses [91]. Disabling MAPK pathway can be a useful approach to inhibit gut inflammation [92]. cGM@CUR-UsNF significantly downregulated the expression of p-JNK/JNK and p-p38/p38 as compared to the VAN group (*p* < 0.05). Consequently, cGM@CUR-UsNF significantly reduced the levels of IFN-γ, TNF-α, IL-1β, and IL-6 compared to the VAN and nGM@CUR-UsNF groups (*p* < 0.05 to 0.01). It is proposed that the improved oral bioavailability and stability of cGM@CUR-UsNF could play a role in the heightened therapeutic effects.

The molecular mechanisms of the anti-inflammatory action of cGM@CUR-UsNF were examined by evaluating its effects on the NF-κB, STAT3, and MAPK signaling pathways in Caco-2 cells, utilizing specific activators and inhibitors (Fig. S11). Exposure to LPS (an NF-κB activator), colivelin (a STAT3 activator), and anisomycin (a MAPK activator) significantly increased the mRNA levels of TNF-α, IL-6, and IL-1β, confirming the activation of these pathways. Treatment with cGM@CUR-UsNF resulted in a significant downregulation of these cytokines when compared to activator-treated groups (*p* < 0.001), demonstrating inhibitory potency similar to that of BAY 11–7082 (NF-κB inhibitor), WP1066 (STAT3 inhibitor), and SB203580 (MAPK inhibitor). The co-treatment of cGM@CUR-UsNF with individual inhibitors did not yield further suppression, indicating that the nanocarrier may target overlapping signaling nodes or function upstream to simultaneously inhibit the phosphorylation of p-p65, p-STAT3, and p-p38 (Fig. S11A–I).

The concurrent activation of all three pathways (LPS + colivelin + anisomycin) significantly enhanced cytokine expression, thereby confirming the crosstalk and synergistic amplification of epithelial inflammation. Co-treatment with cGM@CUR-UsNF effectively normalized the levels of TNF-α, IL-6, and IL-1β, similar to the effects observed with combined inhibitor treatment, suggesting a broad-spectrum anti-inflammatory modulation (Fig. S11J–K). The findings indicate that cGM@CUR-UsNF demonstrates efficacy by simultaneously inhibiting the NF-κB, STAT3, and MAPK pathways, leading to the transcriptional silencing of proinflammatory mediators.

### 3.5 Mechanism of actions of cGM@CUR-UsNF for combating intestinal epithelial barrier dysfunction and gut inflammation

**Fig. 7:**
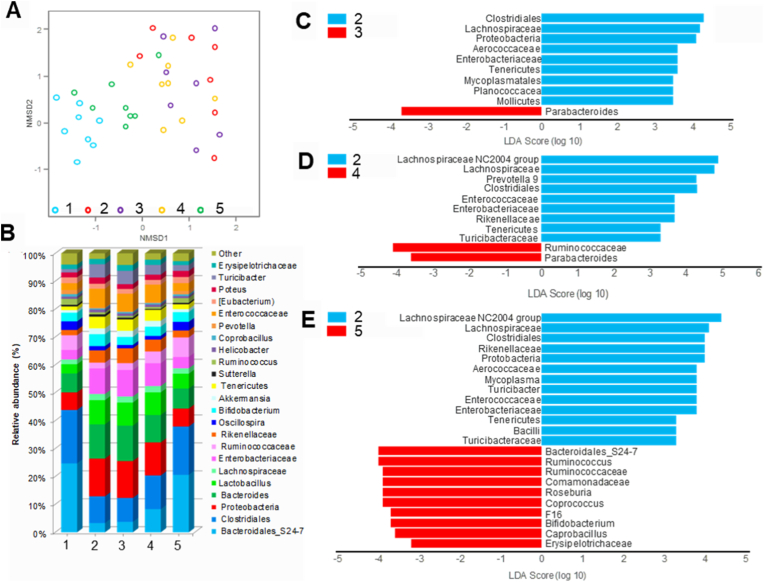
The effect of UsNFs on the microbial composition in VAN-induced mice. (A) NMDS diagram as per Bray–Curtis dissimilarity and (B) the relative abundance of faecal bacterial genera (*n* = 5). Analysis of differences in the microbial taxa shown by LEfSe (LDA) between (C) 2 and 3 groups, (D) 2 and 4 groups, (E) 2 and 5 groups (*n* = 5). 1, CON; 2, VAN; 3, free-CUR; 4, nGM@CUR-UsNF; 5, cGM@CUR-UsNF.

Further investigations were performed to understand how cGM@CUR-UsNF affects the health of the intestines and gut inflammation. It is well-established that changes in gut microbiota caused by VAN, are linked to gut permeability [93], which leads to worse intestinal inflammation-related problems [94]. Restoring GM regulates intestinal epithelial barrier functionalities, gut inflammation, and supplies necessary components for colonocytes, which maintain gut integrity and provide other health benefits to its host [42,[95], [96], [97]]. Using metagenomic 16S rRNA gene sequencing, we examined the changes in GM composition among all the groups, in addition to immunological profiles. The results of NMDS at the operational taxonomic units (OTU) level displayed a strong partitioning between the CON and VAN groups (Fig. 7A). The cGM@CUR-UsNF group displayed a junction area with the CON group in the NMDS map, signifying that cGM@CUR-UsNF restored the VAN-altered gut bacteria to a great extent. Fig. 7B illustrates the aggregated changes in the relative abundance of GM for all groups. The cGM@CUR-UsNF administration considerably raised the relative abundance of *Bacteroidales S_24.7* (6.2-fold), *Clostridiales* (1.8-fold), *Ruminococcaceae* (3.6-fold), *Oscillospira* (2.0-fold) as well as reduced the populations of *Proteobacteria* (52.6 %), *Bacteroides* (41.4 %), *Lactobacillus* (38.4 %), *Enterobacteriaceae* (55.5 %), *Rikenellaceae* (43.7 %), *Tenericutes* (60 %), *Enterococcaceae* (57.6 %) and *Turicibacter* (56.3 %) compared to the VAN group (*p* < 0.05 to 0.001). Hence, cGM@CUR-UsNF treatment could restore VAN-altered GM. The LEfSe analysis was performed to assess the differences in GM alterations among the groups. The histogram of the LDA score showed a clear variance between VAN and cGM@CUR-UsNF (Fig. 7E). The phylum *Protobacteria* and *Tenericutes*, class *Bacilli*, families *Enterococcaceae*, *Rikenellaceae*, and *Aerococcaceae*, and genera *Turicibacter* and *Mycoplasma* were elevated in VAN, free-CUR, and nGM@CUR-UsNF groups (Fig. 7C and D). On the other hand, the genera *Bacteroidales_S24-7*, *Ruminococcus*, *Biffidobacterium*, *Roseburia*, and *Coprococcus* and families *Erysipelotrichaceae* and *Comamonadaceae* increased their abundances in the cGM@CUR-UsNF group (Fig. 7E). The free-CUR increased *Parabacteroides*, while nGM@CUR-UsNF enriched the abundance of *Ruminococcaceae* and *Parabacteroides*.

The heat map-cluster analysis (Fig. S12A) revealed similarity in microbial composition with that of the GM (Fig. 7B). The abundance of *Lactobacillus* and *Turicibacter* in *Firmicutes*; *Bacteroides* and *Rikenellaceae* in Bacteroidetes; *Acinetobacter* and *Pseudomonas* in *Proteobacteria* was significantly higher, while *Bacteroidales* in Bacteroidetes; *Clostridiales* and *Ruminococcaceae* in *Firmicutes*; and *F16* in *TM7* were markedly reduced in the VAN-administered group compared to the CON group. Treatment with cGM@CUR-UsNF brought back the altered microbiota to a significant extent. The elevated levels of *Turicibacter*, *Enterobacteriaceae,* and *Aerococcaceae* were also reduced in the cGM@CUR-UsNF-treated group. Additionally, the levels of *Roseburia* and *Parabacteroides* significantly increased with the use of cGM@CUR-UsNF, which had dropped a lot after treatment with VAN. The qPCR analysis was carried out to measure the abundance of some key bacteria reshaped by cGM@CUR-UsNF (Fig. S12B−G). The cGM@CUR-UsNF remarkably increased the abundance of *Roseburia* and *Parabacteroides* (*p* < 0.001). There were notable increases in the amounts of the bacteria *Lactobacillus*, *Turicibacter*, *Acinetobacter*, and *Pseudomonas* in the VAN group compared to the CON group (*p* < 0.01 to *p* < 0.001). All these significantly decreased by cGM@CUR-UsNF administration (*p* < 0.01 to *p* < 0.001). The abundance of *Bacteroides S_24.7* was markedly reduced in VAN mice (*p* < 0.05), while administration of cGM@CUR-UsNF reversed this reduction (*p* < 0.001). However, free-CUR and nGM@CUR-UsNF did not show any significant restoring effects on the GM.

**Fig. 8:**
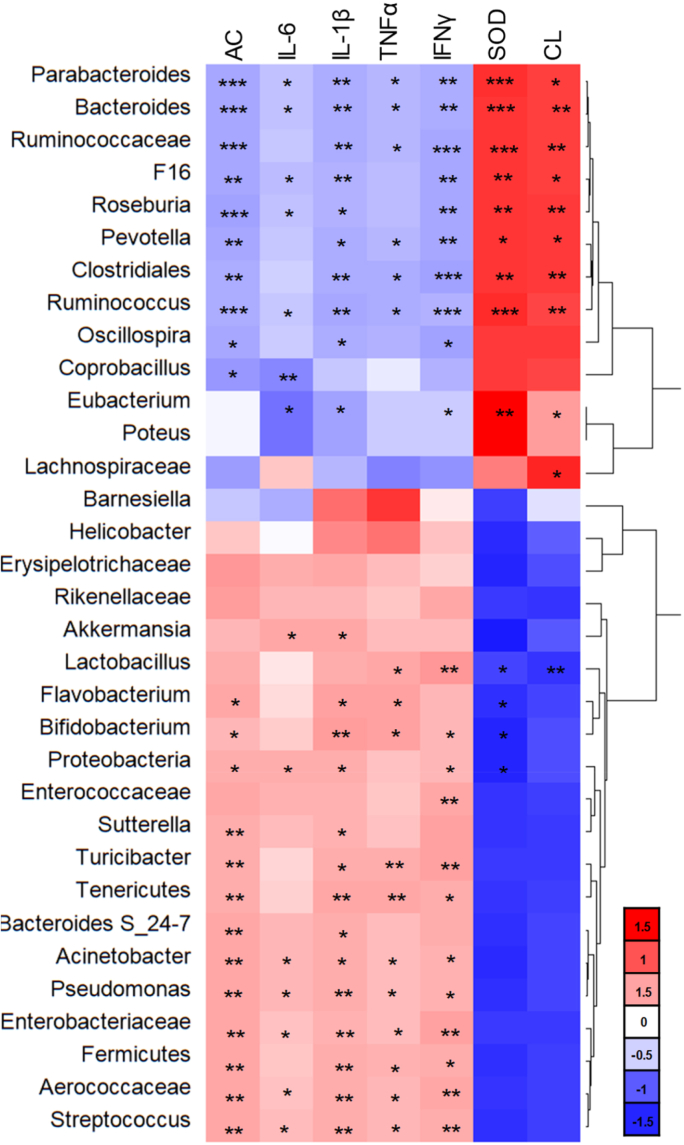
The Spearman correlation analysis between VAN-induced intestine inflammation-related parameters and gut bacteria. CL, colon length; AC, apoptosis^+^ cells. Statistically significant correlations are indicated as *p < 0.05, **p < 0.01 and ***p < 0.001 (*n* = 5).

We next performed a correlation analysis to determine the relationship between the inflammation-related parameters and the GM (Fig. 8). The resultant heat map revealed that the genera *Bacteroides* and *Ruminococcus* and the family *Ruminococcaceae* were significantly improved by cGM@CUR-UsNF administration, which were positively correlated with colon length (*p* < 0.01) and iSOD (*p* < 0.001) and negatively correlated with IFN-γ, TNF-*α*, IL-1β, IL-6, and AC (p < 0.05 to *p* < 0.001). Furthermore, genera such as *Turicibacter*, *Acinetobacter*, *Pseudomonas*, and *Streptococcus* and families *Enterococcaceae* and *Aerococcaceae* were positively correlated with pro-inflammatory cytokines and apoptotic cells in the intestine (*p* < 0.05 to *p* < 0.01). All these were potentially decreased by cGM@CUR-UsNF treatment. These results are in accordance with the other experiments conducted in the present study. The cGM@CUR-UsNF restored the population of beneficial probiotics and reduced the abundance of inflammation-causing gut bacteria, which can suppress VAN-induced gut inflammation and maintain the intestinal cells healthy [10,15,44,98].

**Fig. 9:**
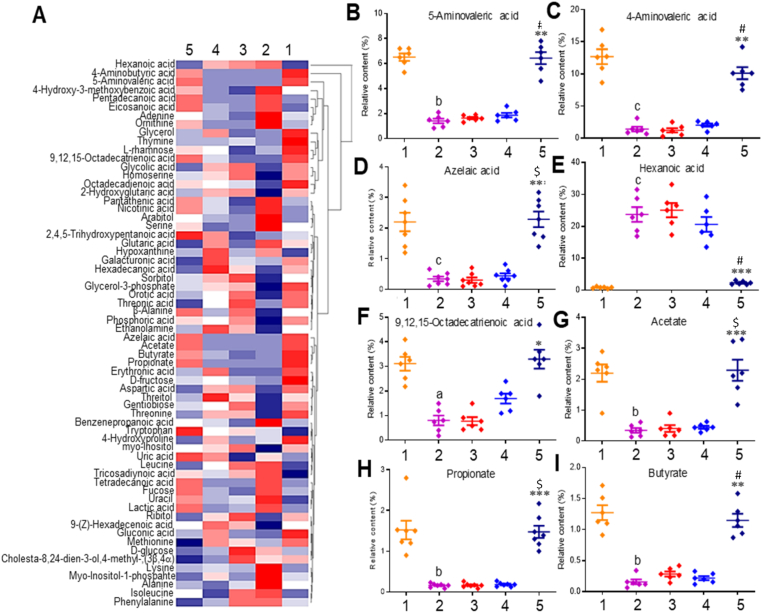
The effect of UsNFs on the compositions of faecal metabolites in VAN-induced mice (*n* = 5). (A) Heat map cluster analysis. (B–I) The relative abundance of specific indicated metabolites. ^a^*p* < 0.05, ^b^*p* < 0.01, ^c^*p* < 0.001 compared to CON group; *p < 0.05, **p < 0.01, ***p < 0.001 compared with VAN group; **^#^***p* < 0.01, **^$^***p* < 0.001 compared with nGM@CUR-UsNF group. 1, CON; 2, VAN; 3, free-CUR; 4, nGM@CUR-UsNF; 5, cGM@CUR-UsNF.

**Fig. 10:**
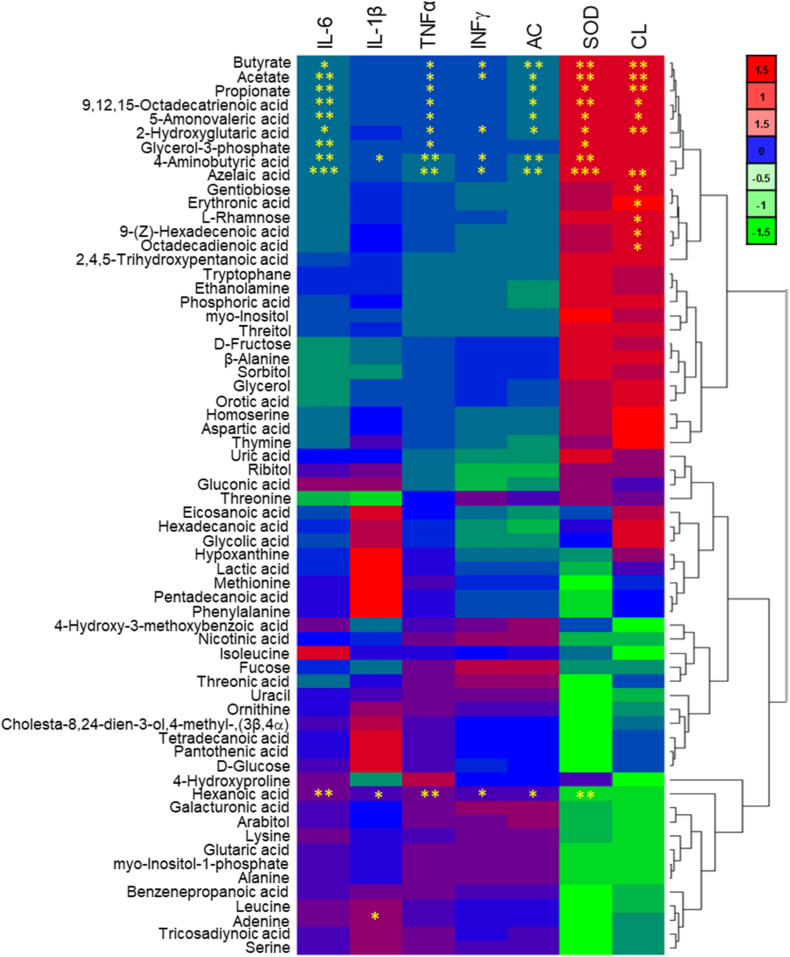
The Spearman correlation analysis between VAN-induced intestine inflammation-related parameters and faecal metabolites of gut bacteria. CL, colon length; AC, apoptosis^+^ cells. Statistically significant correlations are indicated as *p < 0.05, **p < 0.01 and ***p < 0.001.

It is known that the short-chain fatty acids (SCFAs) are major products of GM that maintain gut homeostasis and exert anti-inflammatory actions [99]. CUR could exert its prebiotic effects by restoring the population of SCFA-producing bacteria. We used untargeted GC-MS analysis to examine changes in the metabolites found in the feces of mice and found 64 different substances (Fig. 9A). The detailed information on the characterized metabolites is given in Table S6. The results showed a clear difference between the VAN and CON groups. The cGM@CUR-UsNF-treated group showed a different pattern of metabolite production in the heat map compared to the VAN-induced mice, indicating that giving cGM@CUR-UsNF helped to partially fix the metabolic changes caused by VAN. Previous studies have shown that higher amounts of acetate, propionate, and butyrate can help improve the gut barrier and reduce inflammation in the gut [100]. The high level of hexanoic acid could potentially disrupt gut integrity by altering the microbiome, promoting inflammation, or directly damaging the gut lining [101]. Moreover, azelaic acid is an anti-inflammatory metabolite of the gut bacteria [102]. A significant reduction in the content of 5-aminovaleric acid (from 6.52 % to 1.41 %), 4-aminovaleric acid (from 12.7 % to 1.4 %), azelaic acid (from 2.2 % to 0.34 %), 9,12,15-octadecatrienoic acid (from 3.11 % to 0.8 %), acetate (from 1.52 % to 0.16 %), propionate (from 1.3 % to 0.16 %), butyrate (from 1.1 % to 0.14 %), and enhancement in hexanoic acid (from 23.7 % to 0.81 %) was observed in the VAN-induced group compared to the CON group (*p* < 0.05 to *p* < 0.001) (Fig. 9B−I). The cGM@CUR-UsNF treatment significantly enhanced the contents of acetate, propionate, and butyrate, and decreased the content of hexanoic acid compared to the VAN group (*p* < 0.01 and *p* < 0.001). The correlation analysis clearly showed that the contents of acetate, propionate, butyrate, and azelaic acid, which increased by cGM@CUR-UsNF, were positively correlated with colon length (*p* < 0.01) and iSOD (*p* < 0.01 and *p* < 0.001) but negatively correlated with IFN-γ, TNF-*α*, IL-1β, IL-6, and apoptotic cells (*p* < 0.05 to *p* < 0.001) (Fig. 10). Based on our metabolite study and previous research, we can say that cGM@CUR-UsNF helped increase the number of good bacteria that produce antiinflammatory SCFAs in the intestine, which might help protect the gut lining and reduce inflammation caused by VAN.

**Fig. 11:**
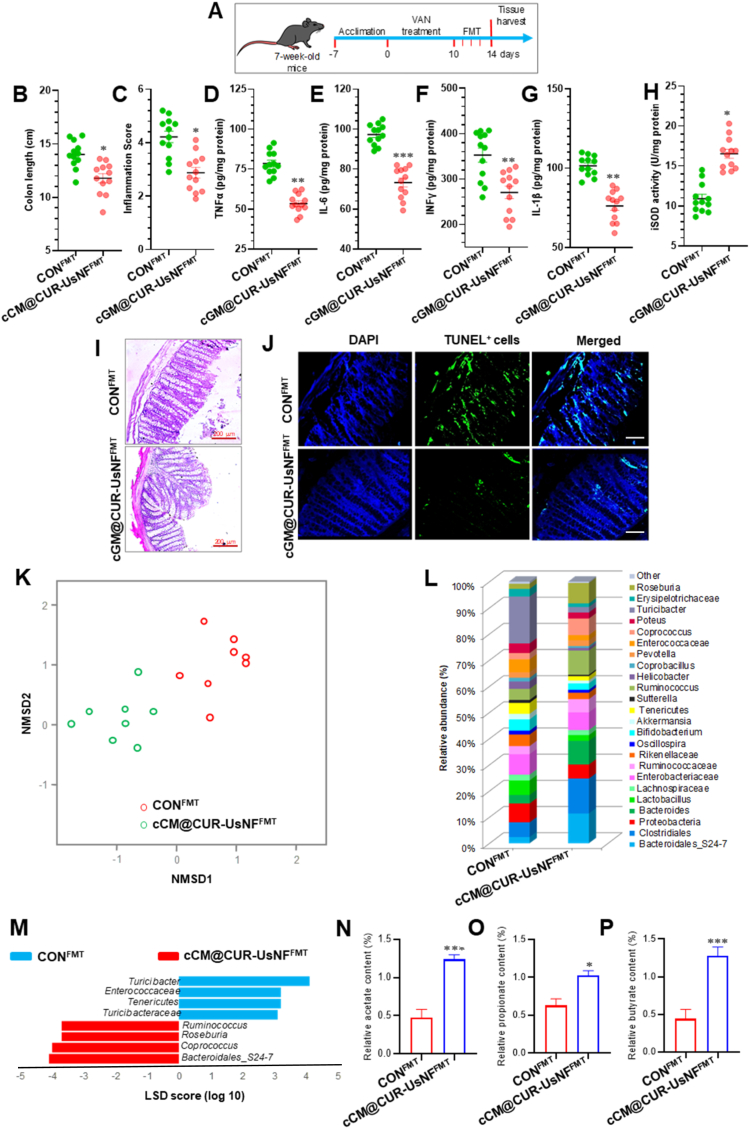
The effect of cGM@CUR-UsNF^FMT^ on inflammatory parameters and oxidative stress in VAN-induced mice (*n* = 5). (A) FMT experimental design. (B) Inflammation score. (C) Colon length (CL). (D) iSOD activity. (E) TNF-α. (F) IL-6. (G) INF-γ. (H) IL-1β. (I) H&E-stained colon sections. (J) TUNEL analysis. Scale bars 50 μm. Impact of cGM@CUR-UsNF^FMT^ on the composition of gut microbiota and metabolites in VAN-induced mice (*n* = 5). (K) NMDS diagram as per Bray–Curtis dissimilarity; (L) the relative abundance of faecal bacterial genera; analysis of differences in the microbial taxa shown by LEfSe (LDA) between (M) CON^FMT^ and cGM@CUR-UsNF^FMT^ groups. Relative abundance of specific metabolites: (N) acetate; (O) propionate; (P) butyrate. *p < 0.05; **p < 0.01; ***p < 0.001 compared to CON^FMT^ group.

We next performed fecal microbiota transplant (FMT) experiments using mice that had been treated with VAN and either received fecal microbiota from cGM@CUR-UsNF-treated mice (cGM@CUR-UsNF^FMT^) or from vehicle-treated mice (CON^FMT^) to check the health benefits of cGM@CUR-UsNF (Fig. 11A). The group that received cGM@CUR-UsNF^FMT^ showed a notable shortening of colon length compared to the CON^FMT^ group (*p* < 0.05) (Fig. 11B). Administration of cGM@CUR-UsNF^FMT^ also contributed to greater reductions in inflamed scores and the levels of TNF-α, IL-6, INFγ, and IL-1β in the colonic tissues, compared to the CON^FMT^ group (Fig. 11C−G; *p* < 0.05 to *p* < 0.001). The higher level of iSOD was also observed in the cGM@CUR-UsNF^FMT^-treated group (Fig. 11H; *p* < 0.05). Also, the transplant of cGM@CUR-UsNF^FMT^ reduced the signs of intestinal inflammation more effectively than the CON^FMT^ group, as shown by the lower histological score (Fig. 11I) and AC (Fig. 11J) in the colon tissues. We next analyzed the composition of GM (Fig. 11K). The NMDS analysis showed that the gut bacteria composition changed between mice treated with cGM@CUR-UsNF^FMT^ and those treated with CON^FMT^, suggesting that cGM@CUR-UsNF^FMT^ influenced the gut bacteria in a noticeable way. The abundance of genus *Bacteroides_S24.7* in Bacteroidetes and *Roseburia*, *Ruminococcus,* and *Coprococcus* in Firmicutes was significantly enhanced by cGM@CUR-UsNF^FMT^ (Fig. 11L and M). The levels of acetate, propionate, and butyrate were significantly increased by cGM@CUR-UsNF^FMT^ compared to CON^FMT^ (Fig. 11N−P; *p* < 0.05 to *p* < 0.001). Overall, cGM@CUR-UsNF^FMT^ ameliorated VAN-induced inflammation and barrier leakiness in the mice colon more strongly than CON^FMT^. Along with our treatment and FMT experiments, cGM@CUR-UsNF^FMT^ also helped increase the good bacteria that produce SCFAs and boosted the production of antiinflammatory SCFAs, showing the healing benefits of cGM@CUR-UsNF by restoring GM.

Collectively, these results suggest that cGM@CUR-UsNF exhibited significant therapeutic efficacy in addressing VAN-induced intestinal epithelial barrier dysfunction and gut inflammation in mice. The treatment successfully inhibited body weight loss and spleen enlargement, demonstrating systemic anti-inflammatory effects, while maintaining normal liver indices. cGM@CUR-UsNF significantly enhanced intestinal barrier integrity through the restoration of tight junction proteins (occludin, ZO-1, claudin-3), a reduction in epithelial apoptosis, and a downregulation of caspase-3 activity.

At the molecular level, it inhibited the NF-κB/STAT3 and MAPK (JNK/p38) signaling pathways, resulting in a decrease of pro-inflammatory cytokines (TNF-α, IL-6, IFN-γ, and IL-1β). Analysis of gut microbiota indicated that cGM@CUR-UsNF effectively restored dysbiosis induced by VAN, significantly enhancing beneficial short-chain fatty acid (SCFA)-producing taxa, including *Roseburia*, *Bacteroidales* S24-7, *Ruminococcus*, and *Coprococcus*, while inhibiting pro-inflammatory *Proteobacteria* and *Turicibacter*.

Metabolomic profiling revealed elevated levels of acetate, propionate, and butyrate are important anti-inflammatory SCFAs alongside reduced hexanoic acid, which correlates with diminished oxidative stress and inflammation. Fecal microbiota transplantation from cGM@CUR-UsNF-treated mice further confirmed its microbiota-mediated protective effects against intestinal injury and inflammation. The results indicate that cGM@CUR-UsNF provides diverse therapeutic advantages by synergistically restoring the gut barrier, modulating inflammation, and rebalancing GM via SCFAs production, presenting an effective approach for addressing antibiotic-induced intestinal epithelial barrier dysfunction and gut inflammation.

### 3.6 Biocompatibility of cGM@CUR-UsNF

**Fig. 12:**
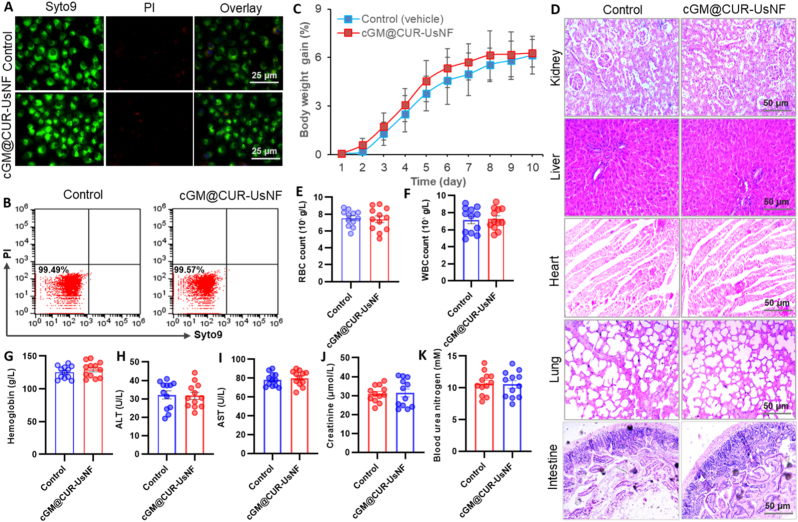
Biocompatibility of cGM@CUR-UsNF (*n* = 12). (A and B) Cell viability of Caco-2 cells. (C) Body weight gain, (D) histology of major mice organs, (E) RBC count, (F) WBC count, (G) hemoglobin, (H) ALT, (I) AST, (J) creatinine, and (K) blood urea nitrogen.

The biocompatibility and safety of cGM@CUR-UsNF were assessed to determine its suitability for clinical application. The cGM@CUR-UsNF is an herbal nanomaterial. Consequently, cGM@CUR-UsNF is expected to exhibit high biocompatibility, devoid of any deleterious side effects in the diagnosis and treatment of VAN-induced intestinal epithelial failure, gut inflammation, and GM dysbiosis. As anticipated, cGM@CUR-UsNF did not influence the viability of Caco-2 cells at 5 mg mL^−1^ over 48 h of incubation, measured by fluorescence microscopy (Fig. 12A) and flow cytometry (Fig. 12B) using Live/Dead staining kit (Thermo Fisher, USA). exhibiting remarkable biocompatibility at the cellular level. The safety and biocompatibility of cGM@CUR-UsNF were further validated in mice following a 10-day dose of 50 mg kg^−1^, with the animals being euthanized on the 11th day to assess the effects of cGM@CUR-UsNF on the structure and function of key organs and the hematological system. The cGM@CUR-UsNF did not affect the body weight of mice (Fig. 12C) or the morphology of key organs (kidney, liver, heart, lung, and intestine) (Fig. 12D). The analysis of standard blood tests and associated liver and kidney function indices revealed that cGM@CUR-UsNF did not impact blood cells (including white blood cells, red blood cells, and hemoglobin), alanine aminotransferase, aspartate aminotransferase, creatinine, and blood urea nitrogen (Fig. 12E–K). These results shown that cGM@CUR-UsNF displayed excellent biocompatibility and safety in both *in vitro* and in vivo models that could be used for clinical application [103].

## 4 Conclusion

Herein, we prepared a negatively charged cGM by using TEMPO to oxidize sugar units of galactomannan at the C-6 position, with the goal of creating a very stable and ultrasmall oral nanoformulation of CUR. The formation process was facilitated by hydrogen bonding interactions between the phenolic hydroxyl groups of CUR and the carboxyl groups present on cGM. The developed cGM@CUR-UsNF significantly enhanced the stability of CUR in both *in vitro* and in vivo settings, improved its permeability through the Caco-2 cell layer and mucus, and increased CUR oral bioavailability. It was shown that the cGM@CUR-UsNF contributed to restoring intestinal epithelial barrier dysfunction and GM dysbiosis, established in a VAN-induced mouse model. This study presents a stable ultrasmall oral nanoformulation of CUR aimed at restoring gut integrity, which may be utilized to assess co-administration strategies of cGM@CUR-UsNF with targeted probiotics to improve microbiota-modulating efficacy, as well as to conduct long-term biocompatibility and toxicity evaluations in preclinical inflammatory bowel disease models to further confirm translational potential.

## CRediT authorship contribution statement

**Vivek Sharma:** Writing – review & editing, Visualization, Validation, Supervision, Software, Project administration, Methodology, Investigation, Formal analysis, Data curation, Conceptualization. **Prateeksha Prateeksha:** Software, Methodology, Investigation, Formal analysis, Data curation. **Balwant Paliya:** Methodology, Investigation, Formal analysis, Data curation. **Sateesh Gupta:** Software, Investigation, Formal analysis, Data curation. **Sarvendra Singh:** Software, Methodology, Formal analysis, Data curation. **Anand Anunay:** Software, Methodology, Investigation, Formal analysis. **Sushil Agrahari:** Software, Formal analysis, Data curation. **Shailendra Singh:** Writing – review & editing, Software, Formal analysis, Data curation. **Chandana Rao:** Writing – review & editing, Investigation, Formal analysis, Data curation. **Saroj Barik:** Software, Funding acquisition, Formal analysis, Conceptualization. **Brahma Singh:** Visualization, Validation, Supervision, Resources, Project administration, Funding acquisition, Conceptualization.

## Availability of data and materials

The datasets analyzed during the current study are available from the corresponding author upon reasonable request.

## Declaration of competing interest

The authors declare that they have no known competing financial interests or personal relationships that could have appeared to influence the work reported in this paper.

## Data Availability

Data will be made available on request.
