# Supplementary material for: Engineered an ultrasmall curcumin oral nanoformulation restores intestinal integrity and gut microbiota dysbiosis
Source: Mater Today Bio. 2025 Nov 12;35:102548. doi: 10.1016/j.mtbio.2025.102548 (PMC12702354; doi:10.1016/j.mtbio.2025.102548)
Supplement: Multimedia component 1 [file mmc1.pdf]

Supplementary Data

Engineered an ultrasmall curcumin oral nanoformulation restores intestinal integrity and gut microbiota dysbiosis

Vivek Sharma[1,2#], Prateeksha Prateeksha[1,3#], Balwant Paliya[1], Sateesh Gupta[1], Sarvendra Singh[1], Anand Anunay[1], Sushil Agrahari[1], Shailendra Singh[4], Chandana Rao[1], Saroj Barik[1], Brahma Singh[1*]

[1]*Herbal Nanobiotechnology Lab, Pharmacology Division, CSIR-National Botanical Research Institute, Lucknow-226001, India*

[2]*Department of Biochemistry and Molecular Biology, Thomas Jefferson University, Philadelphia, PA 19107, United States, United States*

[3]*Key Laboratory of Bioactive Peptides of Yunnan Province, Kunming Institute of Zoology, Chinese Academy of Science, Kunming, Yunnan-650000, China*[3]

[4]*Department of Botany, Institute of Sciences, Banaras Hindu University, Varanasi 221005, India*

[#] V.S. and P.P. contributed equally to this work

*Corresponding author

bn.singh.nbri@csir.res.in; singhbrahmanand99@gmail.com (B. Singh)

Supplementary methods

Distribution of UsNFs in the GIT. The C57BL/6J mice were divided into four groups and fasted for 12 h with free access to water. To perform imaging, DiR-loaded UsNFs of CUR were prepared. Then, free-DiR, DiR@cGM-UsNF, and Dir@nGM-UsNF were respectively orally administrated at the dose of 2 mg/kg DiR. The mice were sacrificed at 0.5, 2, 6, and 12 h postadministration. The stomachs and intestines were isolated from mice and DiR distribution was detected by measuring the fluorescence signals using a fluorescence microscope (Leica DFC7000T, Wetzlar, Germany).

FITC-dextran permeability assay. Seven-week-old C57BL/6J mice were fasted for 4 h and administered 4.4 kDa FITC–dextran at a dosage of 0.6 g/kg in PBS. Blood samples were collected at 4 h post-gavage, centrifuged at 2,000 × g for 10 min at 4 °C, and the serum was stored at −80 °C, shielded from light exposure. Fluorescence (Ex 485 nm / Em 528 nm) was measured. Standards (0.1–100 µg/mL) and QC samples in each plate confirmed linearity ($R^2 \geq 0.99$), with a lower limit of quantification of 0.1 µg/mL, a limit of detection of approximately 0.05 µg/mL, and intra/inter-assay coefficient of variation of less than 15%. Serum FD4 concentrations were determined post-background subtraction and subjected to statistical analysis.

PK study. Male C57BL/6J mice weighing between 22–25g were subjected to an overnight fast before the experiments while having unrestricted access to water. The mice were divided into three groups randomly, and treated with free-CUR, nGM@CUR-UsNF, and cGM@CUR-UsNF at a dose of 50 mg/kg CUR *via* oral gavage. The samples were dissolved in ultrapure water. At different time points, blood samples (500 µL) were from the aortaventralis into heparinized microtubes and samples were centrifuged at 5000 rpm for 10 min at 4 °C. The supernatant was collected into tightly sealed plastic tubes and stored at −20 °C until it could be analyzed using LC–MS/MS [1].

In vivo experiments. Animals were freely allowed to access the sterilized drinking water containing VAN (20 mg/ml) for 10 days, followed by daily oral gavage of 100 µL sterilized water supplemented with or without 50 mg/kg of cGM@CUR-UsNF for another 10 days. Control animals received sterilized drinking water. The average water quantity for consumption was ~4 ml per mouse per day. The water bottles were changed once daily to provide fresh VAN and drugs. Animals were divided into five groups which are as follows: (1) CON group: sterilized drinking water for 20 days; (2) VAN group: 20 mg/ml VAN in drinking water for 10 days, followed by sterilized drinking water for another

10 days; (3) Free-CUR group: 20 mg/ml VAN for 10 days, followed by daily oral treatment of 50 mg/kg free-CUR for 10 days; (4) nGM@CUR-UsNF group: 20 mg/ml VAN for 10 days, followed by daily oral treatment of 50 mg/kg nGM@CUR-UsNF for 10 days; (5) cGM@CUR-UsNF group: 20 mg/ml VAN for 10 days, followed by daily oral treatment of 50 mg/kg cGM@CUR-UsNF for 10 days. On day 21, body weight was recorded, and the faeces were taken aseptically and kept at −80 °C for future study. Upon euthanization in CO_2 chambers, the colon, ileum, liver, and spleen were harvested. Colon length was measured. We weighed the liver and spleen to estimate organ indices. A 4.5 mm portion of the mid-colon was washed and then preserved in 10% (v/v) formalin. For histological examination, the mid-colon portion was embedded, sectioned, and stained with H&E. The degree of lymphocyte infiltration and alterations in the crypt structure and luminal surface were quantified. We evaluated the histological score following the method reported earlier [2]. The remaining colon and ileum tissues were snap-frozen in liquid nitrogen and stored at −80 °C before analysis.

FMT experimentation. The fresh faecal pellets of the CON and cGM@CUR-UsNF groups were collected on days 19, 20, and 21. The collected faecal pellets from each group were homogenized and re-suspended in PBS (50 mg feces/ml). We centrifuged the mixtures at 150×g for 2 min and filtered the obtained supernatant using a Whatman Grade 1 filter (11 μm) before using it for FMT experiments. After 10-days administration of VAN (20 mg/ml) in drinking water to 7-week-old female C57BL/6 mice, the mice were subjected to daily oral gavage with 100 µl of CON^{FMT} and $cGM@CUR\text{-}UsNF^{FMT}$ for another 10 days. On day 21, mice were sacrificed and used for physical and biochemical analysis. The Animal Ethics Committee of the Institute approved these studies (1732/GO/Re/S/13/CPCSEA).

References

[1] N.M. Khalil, T.C.F.d. Nascimento, D.M. Casa, L.F. Dalmolin, A.C.d. Mattos, I. Hoss, M.A. Romano, R.M. Mainardes, Pharmacokinetics of curcumin-loaded PLGA and PLGA–PEG blend nanoparticles after oral administration in rats, Coll. Surf. B: Biointer., 101 (2013) 353-360.
[2] X.X. Guo, Y. Xu, R.X. Geng, J. Qiu, X.Y. He, Curcumin alleviates dextran sulfate sodium-induced colitis in mice through regulating gut microbiota, Mol. Nutr. Food. Res., (2022) e2100943.

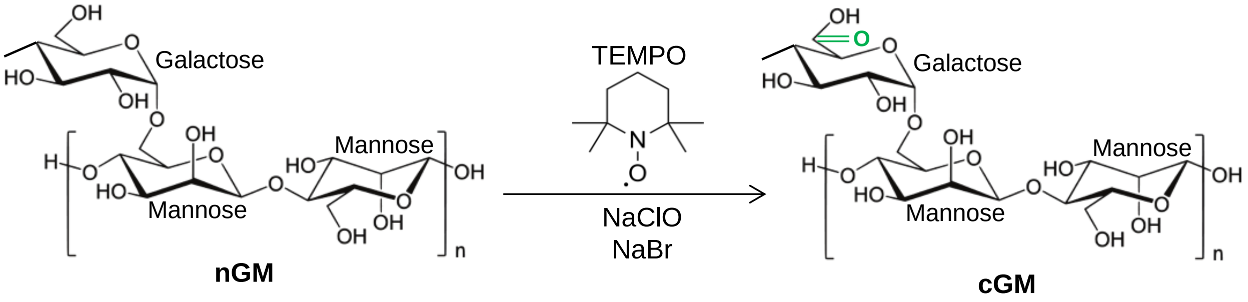

Fig. S1. Schematic illustration of the TEMPO-mediated oxidation of galactomannan.

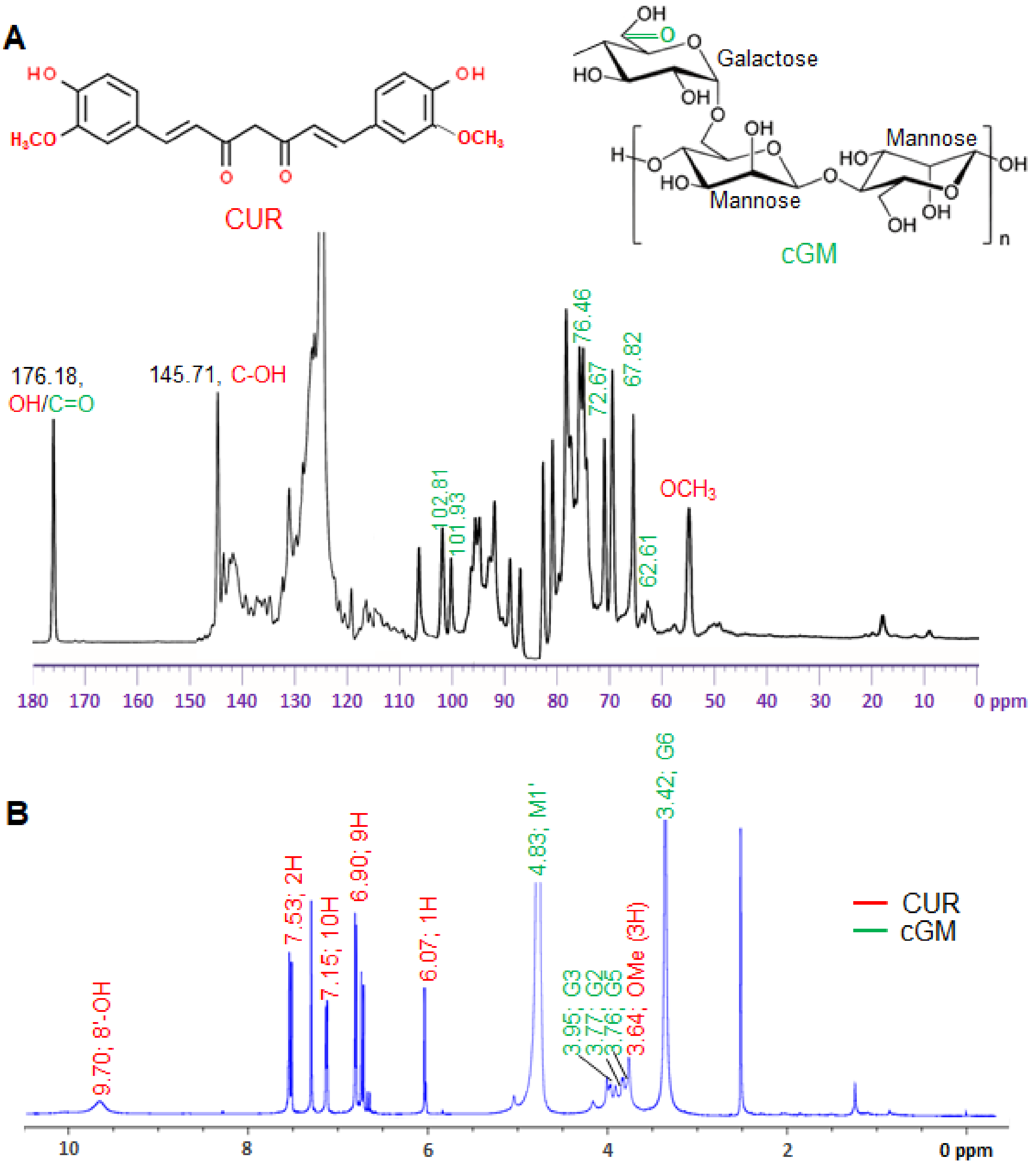

Fig. S2. (A) The ^{13}C-NMR spectra of cGM@CUR-UsNF and (B) ^{1}H NMR spectra of cGM@CUR-UsNF.

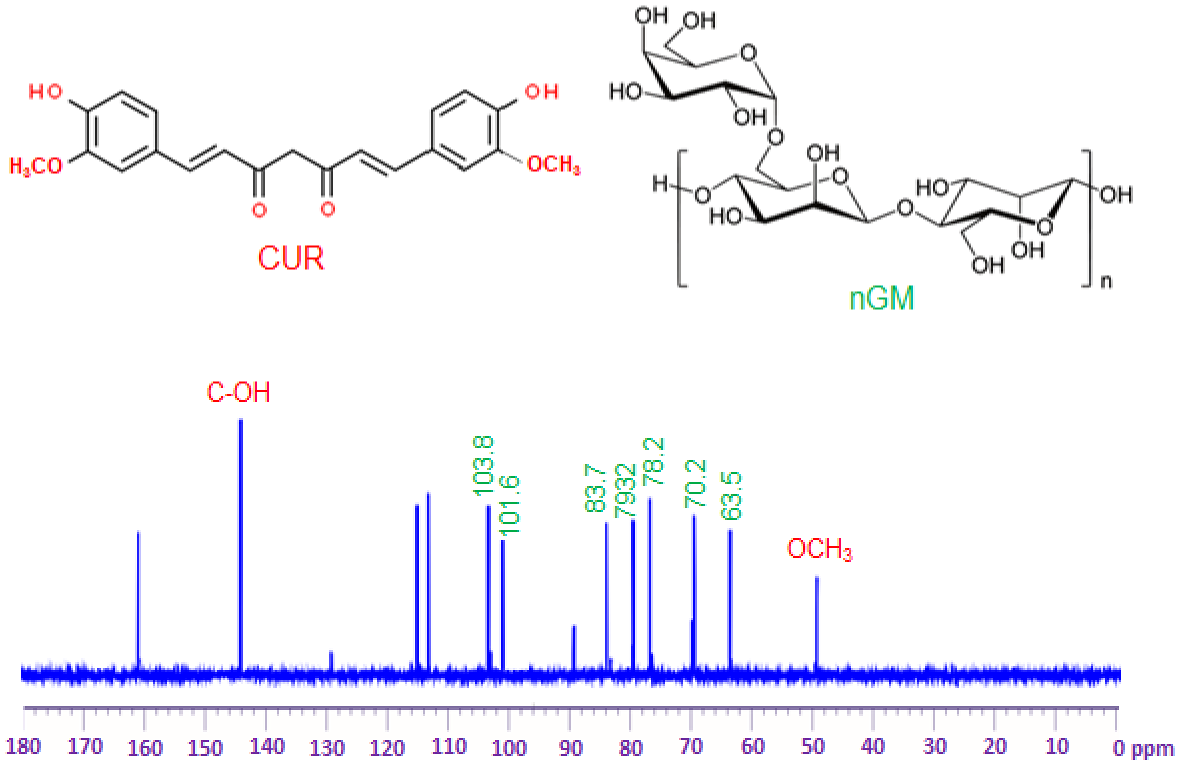

Fig. S3. The ^{13}C-NMR spectra of nGM@CUR-UsNF.

A cGM–COOH + HO–Ar– ⇌ cGM–COOH···HO–Ar–CUR

···, hydrogen bonding
Ar, aromatic ring

B

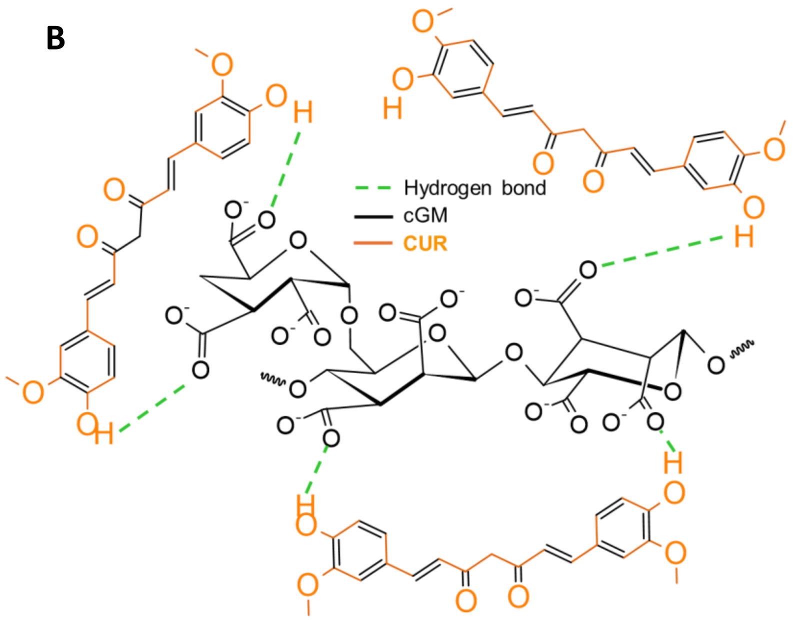

Fig. S4. (A) Reaction equation of the binding of CUR to cGM through non-covalent hydrogen bonding. (B) Chemical interaction mechanism between CUR and cGM.

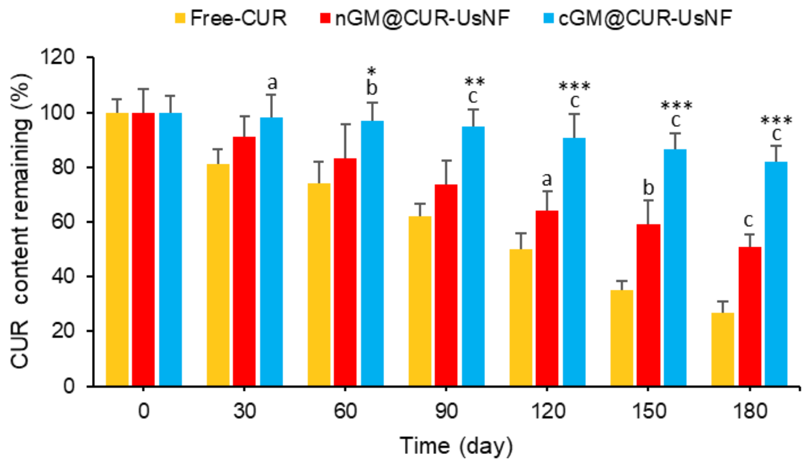

Fig. S5. The remaining CUR content in free-CUR, nCM@CUR-UsNF and nCM@CUR-UsNF within 180 days (n = 3). $^{a}p < 0.05$, $^{b}p < 0.01$, $^{c}p < 0.001$ compared to free-CUR; $^{*}p < 0.05$, $^{**}p < 0.01$, $^{***}p < 0.001$ compared to nGM@CUR-UsNF.

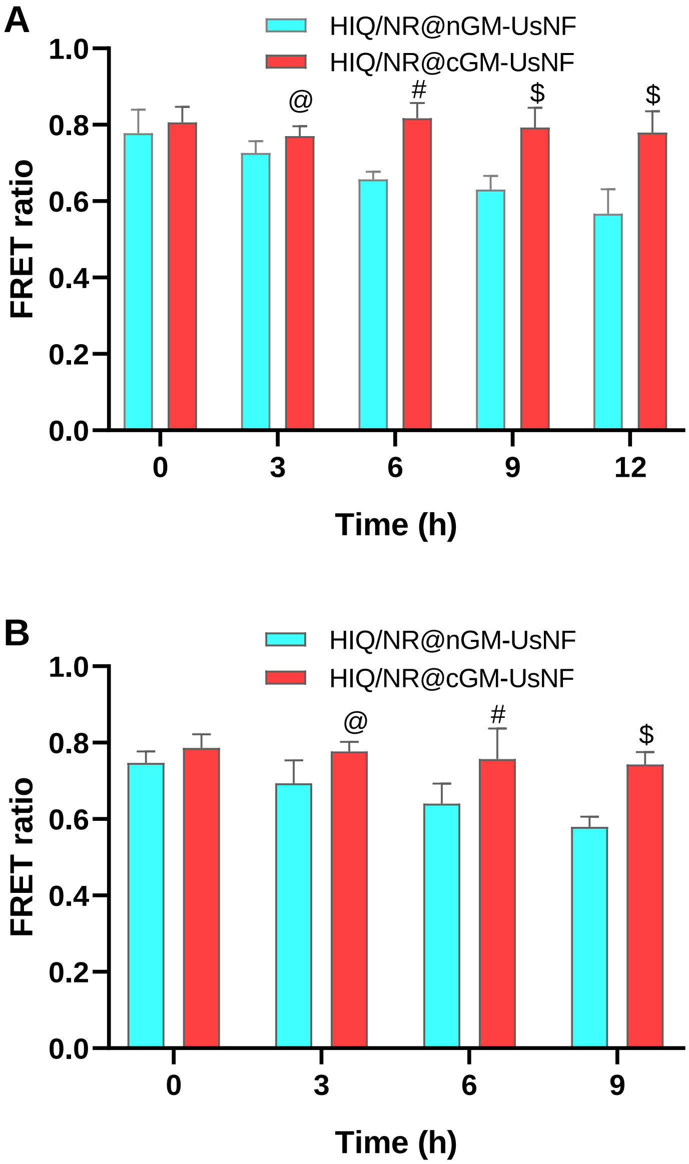

Fig. S6. FRET ratio of UsNFs prepared with HIQ and NR after incubation in (A) SGF and (B) SIF.

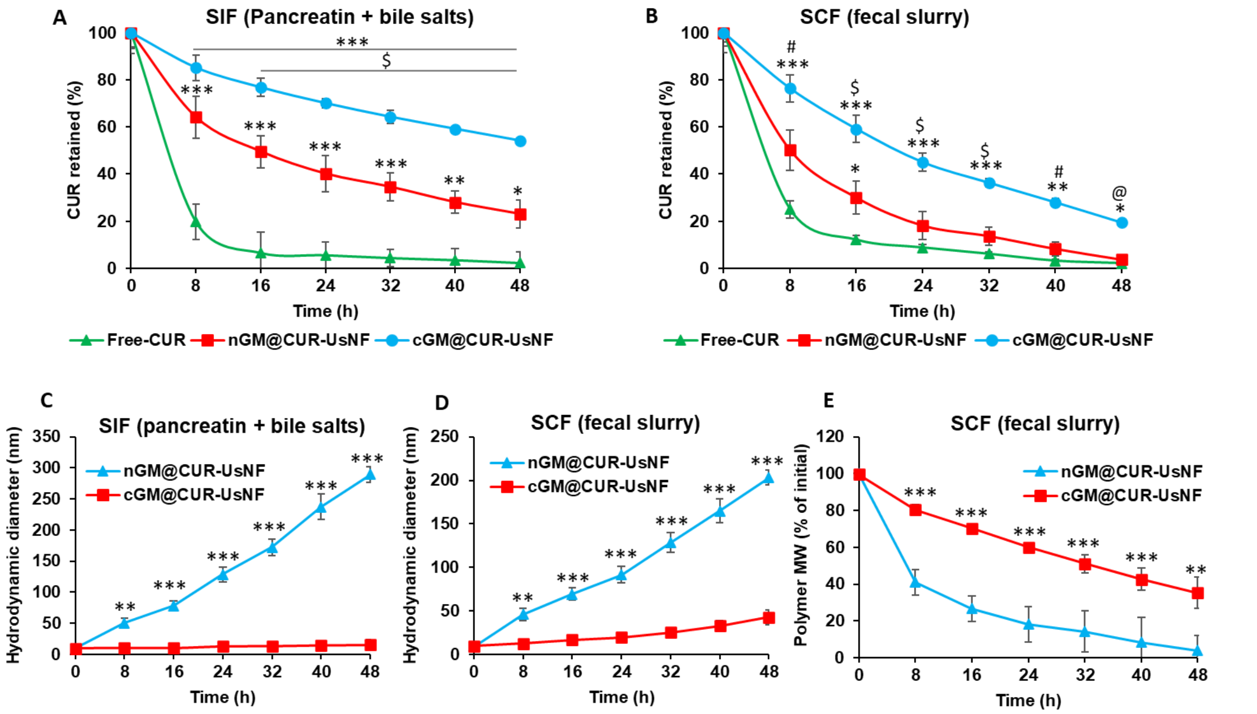

Fig. S7. Stability assessment of UsNFs after incubation in SIF and SCF. CUR was retained in (A) SIF with pancreatin + bile salts and (B) SCF with fecal slurry. Hydrodynamic diameter of UsNFs in (C) SIF with pancreatin + bile salts and (D) SCF with fecal slurry. (E) Polymer molecular weight (MW) retained in SCF with fecal slurry (n = 6). $^{*}p < 0.05$, $^{**}p < 0.01$, $^{***}p < 0.001$ compared with free-CUR group; $^{@}p < 0.05$, $^{\#}p < 0.01$, $^{\$}p < 0.001$ compared with nGM@CUR-UsNF group.

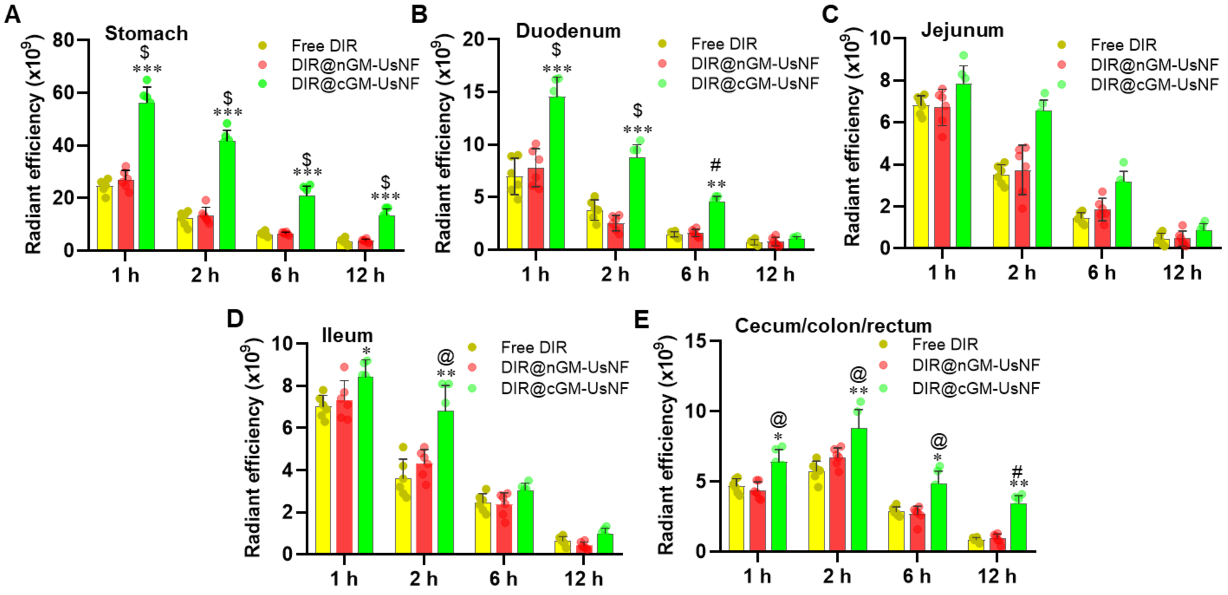

Fig. S8. Retention of UsNFs in the GIT. Fluorescent signals of free DiR, DiR@nGM-UsNF, and DiR@nGM-UsNF in the GIT at various time points after oral administration (n = 6). $^{*}p < 0.05$, $^{**}p < 0.01$, $^{***}p < 0.001$ compared with free-DIR group; $^{@}p < 0.05$, $^{\#}p < 0.01$, $^{\$}p < 0.01$ compared with DIR@nGM-UsNF group

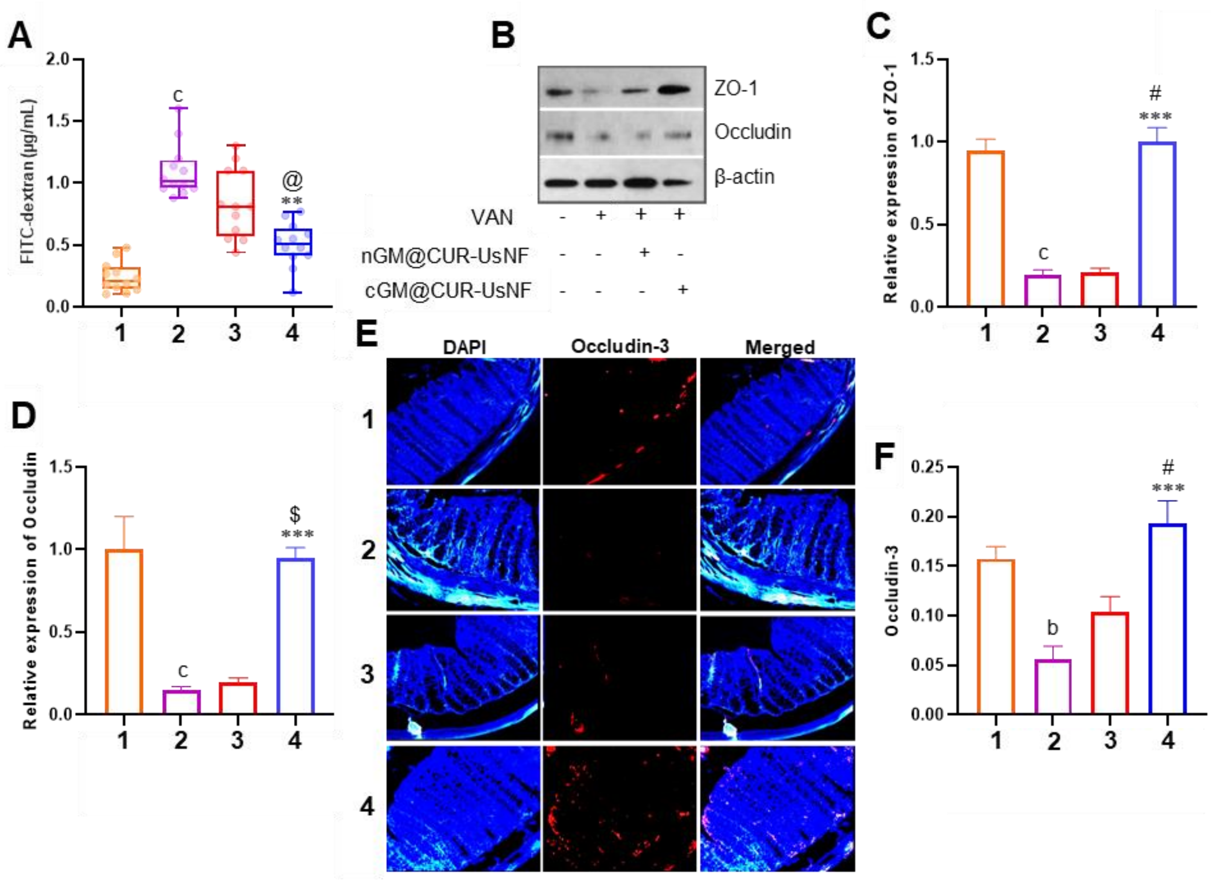

Fig. S9. The effect of UsNFs on VAN-impaired gut tight junction barrier in the colonic tissues (n = 12). (A) FITC-dextran level. (B) Immunoblotting analysis of ZO-1 and occludin proteins in the lysate of colonic tissues. (C) relative expression of ZO-1. (D) relative expression of occluding. (E) images of immunohistochemistry of claudin-3. (F) quantification of claudin-3 expression. $^{b}p < 0.01$, $^{c}p < 0.001$ compared to CON group; $^{**}p < 0.01$, $^{***}p < 0.001$ compared with VAN group; $^{@}p < 0.05$, $^{\#}p < 0.01$, $^{\$}p < 0.001$ compared with nGM@CUR-UsNF group. 1, CON; 2, VAN; 3, nGM@CUR-UsNF; 4, cGM@CUR-UsNF.

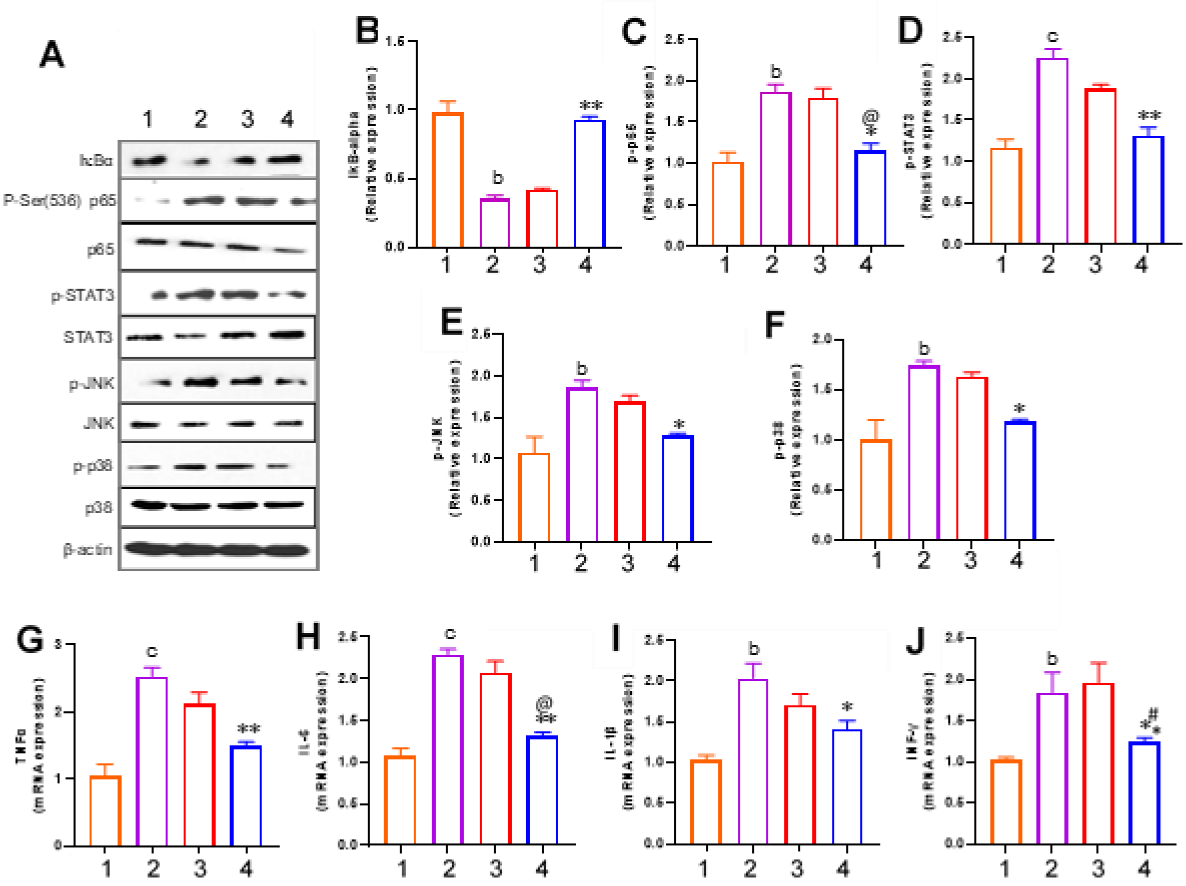

Fig. S10. The effect of UsNFs on VAN-induced gut inflammation in mice. (A) Immunoblotting analysis of indicated proteins: IκB-alpha, p-p65/p65, p-STAT3/STAT3, p-JNK/JNK, and p-p38/p38 in the colonic mice tissues ($n = 6$). Relative expression of (B) IκB-alpha, (C) p-p65, (D) p-STAT3, (E) p-JNK, and (F) p-p38 ($n = 6$). The mRNA expression levels of (G) TNF-α, (H) IL-6, (I) IL-1β and (J) INF-γ in the colon tissue ($n = 6$). $^{b}p < 0.01$, $^{c}p < 0.001$ compared to CON group; $^{*}p < 0.05$, $^{**}p < 0.01$ compared with VAN group; $^{@}p < 0.05$, $^{\#}p < 0.01$ compared with nGM@CUR-UsNF group. 1, CON; 2, VAN; 3, nGM@CUR-UsNF; 4, cGM@CUR-UsNF.

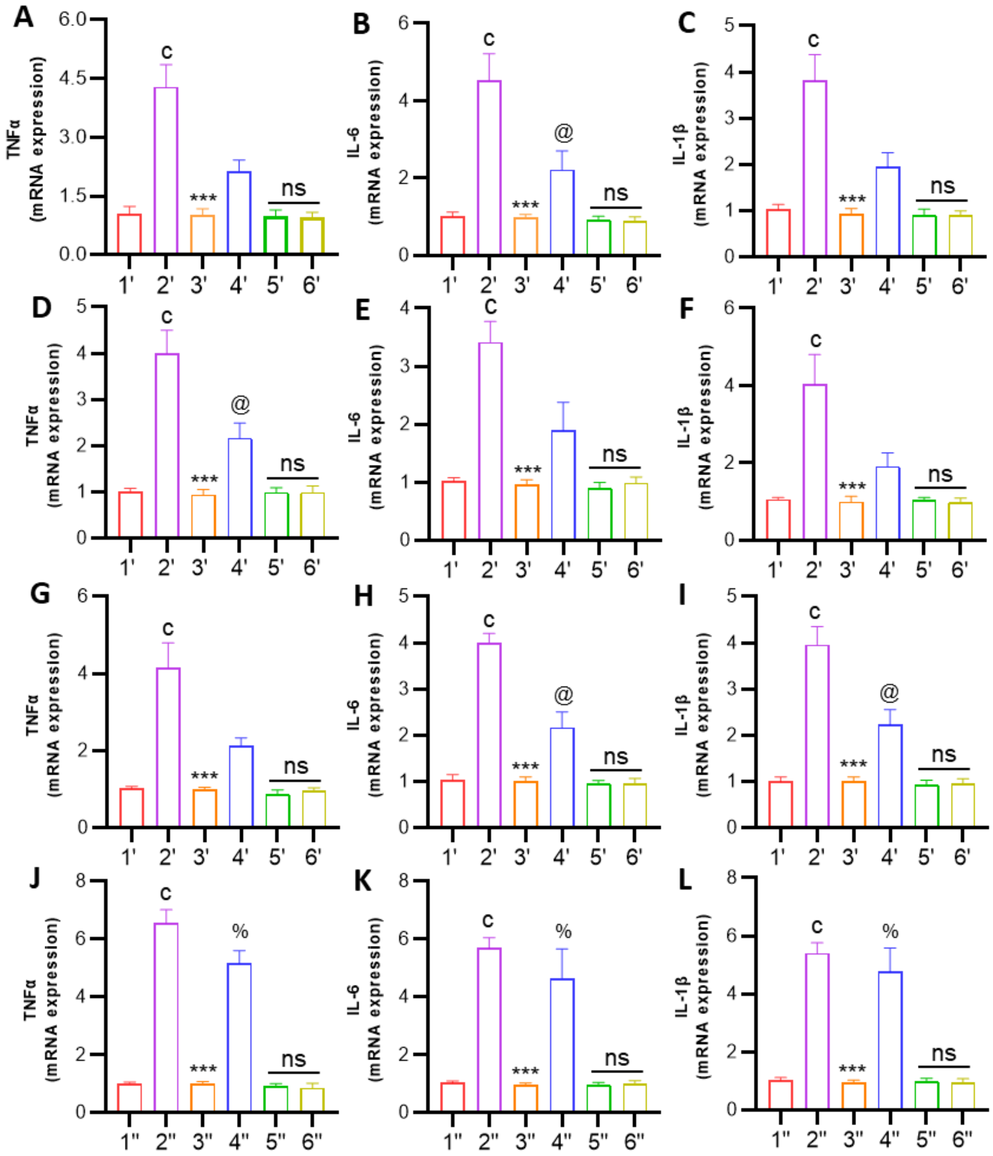

Fig. S11. Elucidation of the mechanisms of actions of cGM@CUR-UsNF in Caco-2 cells. Relative mRNA expression of (A) TNFα, (B) IL-6 and (C) IL-1β in the presence of pharmacological activator (LPS;1 µg/mL) and inhibitor (BAY 11-7082; 5 µM) of NF-κB signalling (n = 12). Relative mRNA expression of (D) TNFα, (E) IL-6 and (F) IL-1β in the presence of pharmacological activator (colivelin; 50 nM) and inhibitor (WP1066; 5 µM) of STAT3 signalling (n = 6). Relative mRNA expression of (G) TNFα, (H) IL-6 and (I) IL-1β in the presence of pharmacological activator (anisomycin; 10 µg/mL) and inhibitor (SB203580; 10 µM) of MAPK signalling (n = 6). **1'** CON; **2'** activator; **3'** cGM@CUR-UsNF; **4'** cGM@CUR-UsNF + activator; **5'** inhibitor; **6'** cGM@CUR-UsNF + inhibitor. Relative mRNA expression of (J) TNFα, (K) IL-6 and (L) IL-1β in the combination of activators-inhibitors of NF-κB, STAT3 and MAPK signalling pathways (n = 12). **1"** CON; **2"** activators (LPS+colivelin+anisomycin); **3"** cGM@CUR-UsNF; **4"** cGM@CUR-UsNF + activators; **5"** inhibitors (BAY 11-7082+WP1066+SB203580); **6"** cGM@CUR-UsNF + inhibitors. $^{c}p < 0.001$ compared to CON group; $^{***}p < 0.001$ compared with VAN group; $^{@}p < 0.05$, $^{\%}p < 0.001$ compared with nGM@CUR-UsNF group. ns, non-significant compared with cGM@CUR-UsNF group.

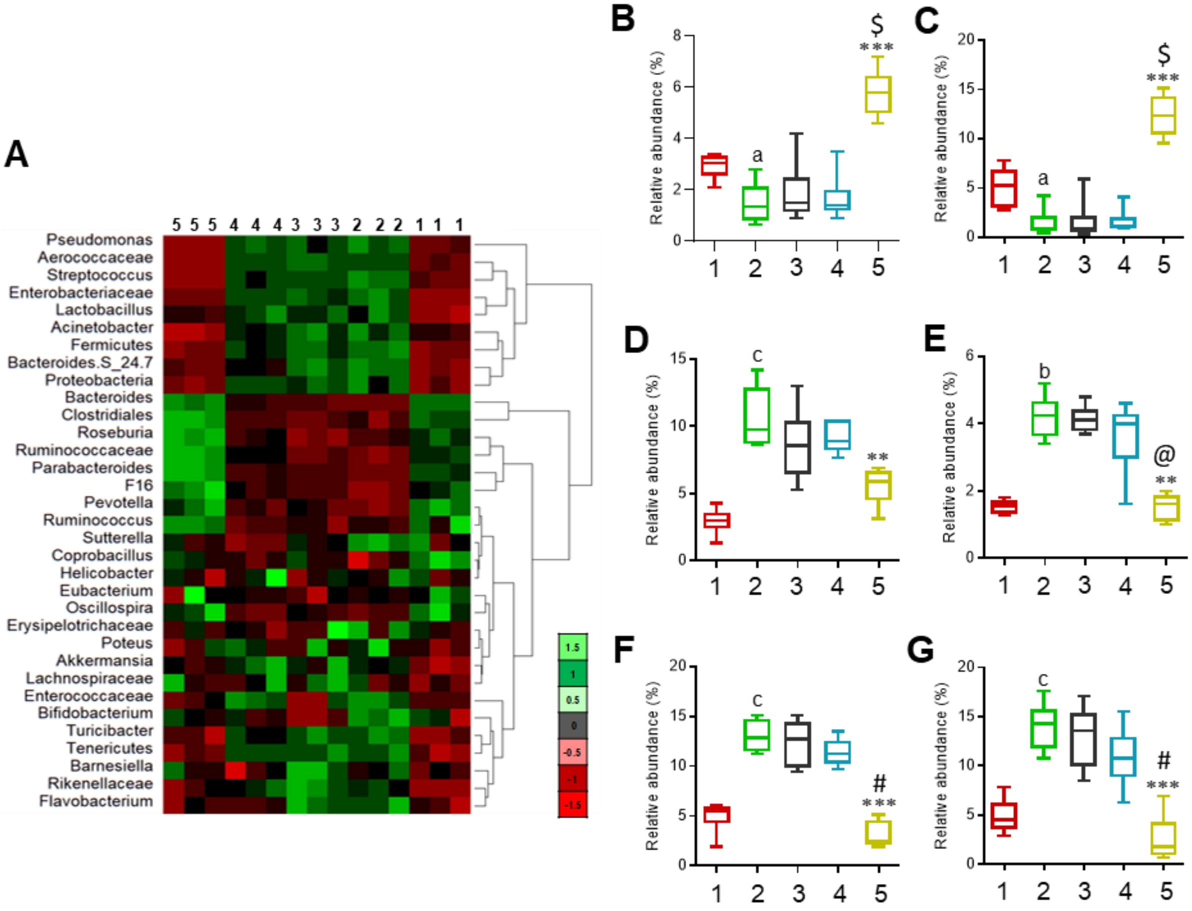

Fig. S12. The effect of UsNFs on the compositions of faecal bacteria in VAN-induced mice (n = 3). (A) Impact of CUR-PS-NPs measured by heat map cluster analysis; the relative abundance of specific bacteria with significant changes by CUR-PS-NPs, namely (B) *Roseburia*; (C) *Parabacteroides*; (D) *Lactobacillus*; (E) *Turicibacter*; (F) *Acinetobacter*; (G) *Pseudomonas*. 1, CON; 2, VAN; 3, free-CUR; 4, nGM@CUR-UsNF; 5, cGM@CUR-UsNF. $^{*}p < 0.05$, $^{**}p < 0.01$, $^{***}p < 0.001$ compared with free-CUR group; $^{@}p < 0.05$, $^{\#}p < 0.01$, $^{\$}p < 0.001$ compared with nGM@CUR-UsNF group.

Table S1. Primers' sequences of cytokine gene used for qRT-PCR

Gene	Primers' sequences
IFN-γ	F:5'-TCAGGTGGCATAGATGTGGAAGAA-3'
	R: 5'-TGGCTTGCAGGATTGTCATG-3'
TNF-α	F: 5'- CCGTCACACTCAGATCATCTTCT-3'
	R: 5'- GCTACGACGTGGGCCACAG-3'
IL-1β	F: 5'- GCAAGTGTTCCTGAACTCAACT-3'
	R: 5'- ATCTTTTGGGCTCCGTCAACT-3'
IL-6	F: 5'- TAGTCGTTCCTACCCCAATTTCC-3'
	R: 5'- TGGTCCTTAGCGACTCCTTC-3'
GAPDH	F: 5'-CATCACTGCCAGCCAGAAGA-3'
	R: 5'-TGAAGTCCCAGGAGACAACC-3'

Table S2. Characteristics of UsNFs

UsNF	LE (%)	LC (%)
nGM@CUR-UsNF	71.34 ± 5.83	11.17± 1.14
cGM@CUR-UsNF	69.81 ± 7.15	10.63 ± 0.73

Data are represented as mean ± SEM (*n* = 3).

Loading efficiency (LE) is the total amount of the CUR that is successfully loaded (or encapsulated) into the cGM relative to the initial amount of the CUR used for loading.

LE (%) = (Amount of CUR initially used/ Amount of CUR loaded into the cGM) ×100

Loading capability (LC) is the maximum amount of a CUR that a cGM can potentially hold or load, often expressed as a weight or volume ratio (e.g., mg of CUR per mg of cGM).

LC (%) = Amount of cGM/ Maximum amount of CUR that can be loaded ×100

Table S3. The stability of UsNFs in SGF and SIF

UsNFs	Average particle size (nm)	Zeta potential (mV)
**SGF** (pH 2.5)		
nGM@CUR-UsNF	361.26 ± 20.41	-0.21 ± 0.05
cGM@CUR-UsNF	17.71 ± 1.02	-34.1 ± 1.33
**SIF** (pH 7.0)		
nGM@CUR-UsNF	119.44 ± 11.14	-7.13 ± 0.52
cGM@CUR-UsNF	14.21 ± 0.24	-26.91 ± 1.18

Data are represented as mean ± SEM (*n* = 6).

Table S4. Physicochemical properties of HIQ/NR loaded UsNFs

Property	HIQ/NR@nGM-UsNF	HIQ/NR@cGM-UsNF
Particle size (nm)	9.22 ± 0.51	9.96 ± 0.42
PDI	0.62 ± 0.013	0.29 ± 0.011
Zeta potential (mV)	0.063 ± 0.002	35.9 ± 3.17
Loading efficiency (%)	HIQ 83.07 ± 4.83	HIQ 85.82 ± 8.62
	NR 81.83 ± 6.93	NR 82.81 ± 7.41

Data are represented as mean ± SEM (*n* = 6).

Table S5. Impact of different doses of UsNFs and free-CUR on body weight and liver weight (*n* = 12)

Group	Treatment	Initial body weight (g)	Final body weight (g)	Liver weight (g)	Rel. liver weight (Liver wt/100 g b.wt)
I	1 ml of 0.8% CMC/kg	20 ± 0.13	21 ± 0.11	1.02 ± 0.02	4.86 ± 0.03
II	25 mg of nGM@CUR-UsNF/kg	19 ± 0.15	20.2 ± 0.14	1.0 ± 0.011	4.95 ± 0.03
III	50 mg of nGM@CUR-UsNF/kg	20.6 ± 0.17	21.1 ± 0.15	1.02 ± 0.02	4.83 ± 0.12
IV	75 mg of nGM@CUR-UsNF/kg	20 ± 0.13	17.1 ± 0.12[a]	1.15 ± 0.025[a]	6.75 ± 0.17[b]
V	25 mg of cGM@CUR-UsNF/kg	20.5 ± 0.14	21.4 ± 0.14	1.06 ± 0.018	4.95 ± 0.14
VI	50 mg of cGM@CUR-UsNF/kg	20.3 ± 0.19	21.5 ± 0.17	1.07 ± 0.031	5.0 ± 0.10
VII	75 mg of cGM@CUR-UsNF/kg	20.5 ± 0.18	17.2 ± 0.21[a]	1.12 ± 0.024[a]	6.51 ± 0.19 [b]
VIII	25 mg of free-CUR/kg	21 ± 0.16	22.02 ± 0.15	1.06 ± 0.036	4.81 ± 0.11
IX	50 mg of free-CUR/kg	20.6 ± 0.2	21.6 ± 0.19	1.05 ± 0.022	4.86 ± 0.23
X	75 mg of free-CUR/kg	20.3 ± 0.22	16.2 ± 0.21[b]	1.19 ± 0.038[b]	7.34 ± 0.16[b]

All the values are expressed as mean ± SEM. [a] to $P < 0.05$ and [b] to $P < 0.01$ compared to respective Normal group-I.

Table S6. Details of detected metabolites in the fecal samples of VAN-induced and treated mice (n = 5)

S. No.	Metabolite	Control *vs* VAN			VAN *vs* free-CUR			VAN *vs* nGM@CUR-UsNF			VAN *vs* cGM@CUR-UsNF		
		p-value	Fold change	VIP	*p*-value	Fold change	VIP	*p*-value	Fold change	VIP	*p*-value	Fold change	VIP
1.	5-Aminovaleric acid	**<0.001**	5.122	1.517	0.006	1.101	1.472	0.021	1.061	1.527	**<0.01**	4.385	1.562
2.	Leucine	0.104	0.284	0.724	0.077	0.704	0.927	0.472	0.678	0.963	0.267	0.339	0.769
3.	Pentadecanoic acid	0.012	0.262	5.270	0.516	0.271	4.73	0.057	0.342	3.752	0.573	0.906	5.110
4.	4-Hydroxyproline	0.009	1.152	0.244	0.572	0.924	0.251	0.163	0.820	0.283	0.163	1.037	0.218
5.	Gluconic acid	0.108	1.185	0.702	0.253	1.119	0.648	0.009	1.140	0.636	0.376	1.084	0.674
6.	9-(Z)-Hexadecenoic acid	0.007	1.515	0.483	0.064	1.102	0.745	0.168	1.008	0.814	0.296	1.525	0.491
7.	Tryptophan	0.513	0.653	0.193	0.279	1.117	0.196	0.372	1.014	0.216	0.168	1.548	0.175
8.	2,4,5-Trihydroxypentanoic acid	0.109	1.159	1.182	0.583	0.778	1.263	0.539	0.829	1.185	0.285	1.645	1.205
9.	Benzenepropanoic acid	0.568	0.454	0.471	0.635	0.368	0.494	0.361	0.345	0.528	0.832	0.671	0.438
10.	4-Aminobutyric acid	**<0.001**	10.21	1.481	0.152	0.998	1.284	0.028	1.014	1.263	0.001	7.742	1.386
11.	Hexadecanoic acid	0.537	1.259	1.213	0.372	1.155	1.283	0.008	1.280	1.158	0.638	0.991	1.274

12.	Tetradecanoic acid	0.014	0.229	1.397	0.583	0.349	1.194	0.003	0.330	1.262	0.682	0.878	1.347
13.	Glycolic acid	0.008	2.320	1.181	0.377	2.144	1.382	0.002	2.505	1.183	0.283	1.072	1.149
14.	Erythronic acid	**<0.05**	4.432	0.289	0.482	1.754	0.183	0.006	1.834	0.175	0.103	1.774	0.297
15.	Thymine	**<0.05**	3.485	1.484	0.084	1.214	1.624	0.265	1.197	1.648	0.009	1.120	1.463
16.	myo-Inositol	0.172	1.974	0.267	0.273	2.179	0.274	0.007	2.314	0.258	0.107	1.939	0.248
17.	Methionine	0.182	1.214	0.719	0.094	1.121	0.704	0.159	1.050	0.752	0.416	0.791	0.736
18.	2-Hydroxyglutaric acid	0.007	3.426	1.115	0.267	3.491	1.183	0.002	3.244	1.273	0.015	2.472	1.064
19.	Isoleucine	0.015	0.821	1.046	0.352	1.058	0.973	0.037	1.071	0.962	0.583	0.845	1.115
20.	Phenylalanine	0.127	0.921	1.056	0.216	0.989	1.063	0.264	1.080	0.974	0.481	0.852	1.064
21.	Ethanolamine	0.126	1.208	1.424	0.301	1.266	1.438	0.381	1.425	1.277	0.385	1.097	1.488
22.	Uric acid	0.418	0.857	0.504	0.417	0.772	0.482	0.862	0.807	0.461	0.173	1.102	0.528
23.	myo-Inositol-1-phosphate	0.681	0.688	1.269	0.384	0.584	1.372	0.375	0.579	1.384	0.741	0.605	1.237
24.	Glycerol	0.009	3.977	1.491	0.104	0.926	1.384	0.047	0.954	1.344	0.274	1.393	1.387

25.	Ribitol	0.377	1.342	0.558	0.341	1.340	0.632	0.009	1.309	0.647	0.026	1.033	0.573
26.	Galacturonic acid	0.229	1.333	1.472	0.245	1.024	1.493	0.032	1.001	1.527	0.583	0.834	1.414
27.	beta-Alanine	0.215	1.196	1.445	0.134	1.217	1.583	0.152	1.356	1.421	0.026	1.410	1.374
28.	Sorbitol	0.316	1.309	0.985	0.547	1.816	0.871	0.002	1.785	0.886	0.268	1.091	1.173
29.	Lactic acid	0.638	0.432	1.464	0.229	0.573	1.273	0.013	0.536	1.362	0.009	1.013	1.364
30.	Pantothenic acid	0.527	0.584	2.192	0.313	0.519	1.962	0.023	0.572	1.782	0.471	0.857	2.138
31.	Threitol	0.114	2.196	0.443	0.026	2.116	0.482	0.015	2.024	0.504	0.023	1.619	0.452
32.	Gentiobiose	0.215	2.016	0.635	0.315	2.357	0.582	0.031	2.399	0.572	0.529	1.461	0.673
33.	Phosphoric acid	0.284	1.189	1.455	0.334	1.212	1.427	0.011	1.225	1.412	0.283	1.190	1.389
34.	Serine	0.573	0.651	2.814	0.199	0.538	2.753	0.016	0.574	2.583	0.628	0.743	2.681
35.	Glutaric acid	0.448	0.863	1.488	0.209	0.708	1.382	0.016	0.720	1.359	0.038	0.598	1.459
36.	Adenine	0.753	0.478	3.810	0.171	0.325	3.942	0.022	0.330	3.874	0.008	0.603	3.612
37.	Uracil	0.691	0.366	1.468	0.136	0.561	1.484	0.412	0.596	1.396	0.275	0.851	1.388
38.	Hypoxanthine	0.484	0.831	1.423	0.736	0.916	1.386	0.133	0.943	1.347	0.651	0.899	1.427

39.	Threonine	0.275	1.464	0.873	0.418	1.275	0.927	0.265	1.236	0.956	0.163	1.114	0.864
40.	Threonic acid	0.118	1.356	1.38	0.219	1.362	1.417	0.329	1.342	1.438	0.683	0.976	1.311
41.	4-Hydroxy-3-methoxybenzoic acid	0.638	0.797	4.82	0.211	0.619	4.742	0.714	0.610	4.817	0.062	0.879	4.742
42.	Lysine	0.558	0.481	1.358	0.422	0.526	1.417	0.051	0.514	1.452	0.474	0.591	1.273
43.	Homoserine	0.127	2.176	1.351	0.068	2.352	1.348	0.056	2.307	1.374	0.052	1.697	1.402
44.	Fucose	0.738	0.661	1.321	0.473	0.580	1.274	0.483	0.571	1.294	0.388	0.944	1.348
45.	Ornithine	0.471	0.487	3.74	0.314	0.453	3.832	0.876	0.993	1.748	0.053	0.835	3.271
46.	Alanine	0.386	0.803	1.282	0.292	0.743	1.296	0.363	0.778	1.237	0.374	0.738	1.247
47.	Arabitol	0.577	0.418	2.830	0.243	0.432	2.763	0.427	0.443	2.692	0.009	1.091	1.552
48.	Glycerol-3-phosphate	0.186	1.970	0.873	0.441	2.032	0.752	0.267	2.003	0.763	0.416	1.381	0.927
49.	Eicosanoic acid	0.263	0.370	3.218	0.149	0.404	3.172	0.054	0.418	3.062	0.117	1.017	3.117
50.	Nicotinic acid	0.628	0.698	1.962	0.414	0.616	2.063	0.034	0.590	2.154	0.517	0.924	1.874
51.	Aspartic acid	0.006	3.521	0.432	0.072	3.075	0.482	0.079	3.463	0.428	0.007	1.584	0.416

52.	Orotic acid	0.482	1.632	1.121	0.167	1.443	1.273	0.769	1.362	1.349	0.136	1.102	1.254
53.	Octadecadienoic acid	0.273	1.684	2.143	0.318	1.285	2.064	0.152	1.345	1.973	0.007	1.445	2.063
54.	Azelaic acid	**<0.01**	7.016	0.372	0.529	0.849	0.351	0.583	0.797	0.374	**<0.01**	6.681	0.364
55.	Hexanoic acid	**<0.001**	0.038	25.81	0.347	1.359	18.26	0.224	1.119	22.16	**<0.001**	0.096	22.64
56.	D-fructose	0.113	2.819	0.649	0.237	1.400	0.537	0.483	1.245	0.604	0.335	1.148	0.752
57.	D-glucose	0.584	0.857	0.762	0.128	1.069	0.815	0.304	1.111	0.785	0.163	0.867	0.684
58.	L-rhamnose	0.008	3.876	0.983	0.562	0.191	0.663	0.311	0.202	0.628	0.006	2.480	0.895
59.	Tricosadiynoic acid	0.443	0.361	0.762	0.012	0.767	0.851	0.069	0.721	0.906	0.247	0.821	0.733
60.	9,12,15-Octadecatrienoic acid	**<0.05**	4.266	0.872	0.432	1.217	0.627	0.811	1.205	0.633	**<0.05**	3.870	0.915
61.	Cholesta-8,24-dien-3-ol, 4-methyl-, (3β,4α)	0.263	1.011	0.864	0.063	1.313	0.748	0.664	1.371	0.716	0.715	0.742	0.879
62.	Acetate	**<0.001**	10.52	0.173	0.411	0.733	0.217	0.257	0.604	0.263	**<0.001**	8.486	0.185
63.	Propionate	**<0.001**	8.941	0.170	0.081	1.530	0.183	0.318	1.451	0.193	**<0.001**	7.439	0.164
64.	Butyrate	**<0.001**	8.333	0.150	0.014	0.168	0.712	0.103	0.176	0.682	**<0.001**	7.101	0.138
